# The miR-6240 target gene *Igf2bp3* promotes myoblast fusion by enhancing myomaker mRNA stability

**DOI:** 10.1186/s11658-024-00650-1

**Published:** 2024-12-05

**Authors:** Yuxin Huang, Wei Wang, Xinhao Fan, Xiaoqin Liu, Weiwei Liu, Zishuai Wang, Yixing Li, Yalan Yang, Zhonglin Tang

**Affiliations:** 1 Kunpeng Institute of Modern Agriculture at Foshan, Agricultural Genomics Institute at Shenzhen, Chinese Academy of Agricultural Sciences, Foshan, 528226 China; 2 Guangxi Key Laboratory of Animal Breeding, Disease Control and Prevention; College of Animal Science and Technology, Guangxi University, Nanning, 530004 Guangxi China; 3 Key Laboratory of Agricultural Animal Genetics, Breeding and Reproduction of Ministry of Education and Key Lab of Swine Genetics and Breeding of Ministry of Agriculture and Rural Affairs, Huazhong Agricultural University, Wuhan, 430070 China; 4 Shenzhen Branch, Guangdong Laboratory for Lingnan Modern Agriculture, Key Laboratory of Livestock and Poultry Multi-Omics of MARA, Agricultural Genomics Institute at Shenzhen, Chinese Academy of Agricultural Sciences, Shenzhen, 518124 China

**Keywords:** *Igf2bp3*, Myobalst fusion, *Mymk*, mRNA stability, miR-6240, Myogenesis

## Abstract

**Background:**

Myoblast fusion plays a crucial role in myogenesis. Insulin-like growth factor 2 mRNA-binding protein 3 (IGF2BP3) functions as an RNA *N*^6^-methyladenosine reader and exerts important roles in various biological processes. While our prior study suggested *Igf2bp3* contributes to myogenesis, its molecular regulatory mechanism is largely unclear.

**Methods:**

Real-time quantitative polymerase chain reaction (RT-qPCR) and western blot were used for gene expression analysis. siRNA and CRISPRi technologies were conducted to knockdown the expression of *Igf2bp3*. CRISPR/Cas9 technology was performed to knockout *Igf2bp3*. The *Igf2bp3* overexpression vector was designed using the pcDNA3.1(+) vector. Immunofluorescence detection was employed for subcellular localization and cell differentiation analysis. Cell Counting Kit-8 (CCK-8) and 5-ethynyl-2′-deoxyuridine (EdU) assays were conducted for cell proliferation and fusion detection. The dual-luciferase reporter assay and RNA immunoprecipitation (RIP) assay were utilized for regulatory mechanism analysis of* Igf2bp3*.

**Results:**

The overexpression of *Igf2bp3* enhances myoblast fusion while knockdown of *Igf2bp3* blocks the formation of myotubes. miR-6240 promotes myoblast proliferation while preventing myoblast differentiation and fusion by targeting the 3′ untranslated rgion (UTR) of *Igf2bp3*. Notably, the impacts of miR-6240 mimics on myoblast proliferation, differentiation, and fusion can be effectively counteracted by the overexpression of *Igf2bp3*. Moreover, our findings elucidate a direct interaction between *Igf2bp3* and the myoblast fusion factor myomaker (*Mymk*). *Igf2bp3* binds to *Mymk* to enhance its mRNA stability. This interaction results in increased expression of *Mymk* and heightened myoblast fusion.

**Conclusions:**

Our study unveils *Igf2bp3* as a novel post-transcriptional regulator of myoblast fusion through the miR-6240/*Mymk* axis, significantly contributing to our understanding of skeletal muscle development.

**Graphical Abstract:**

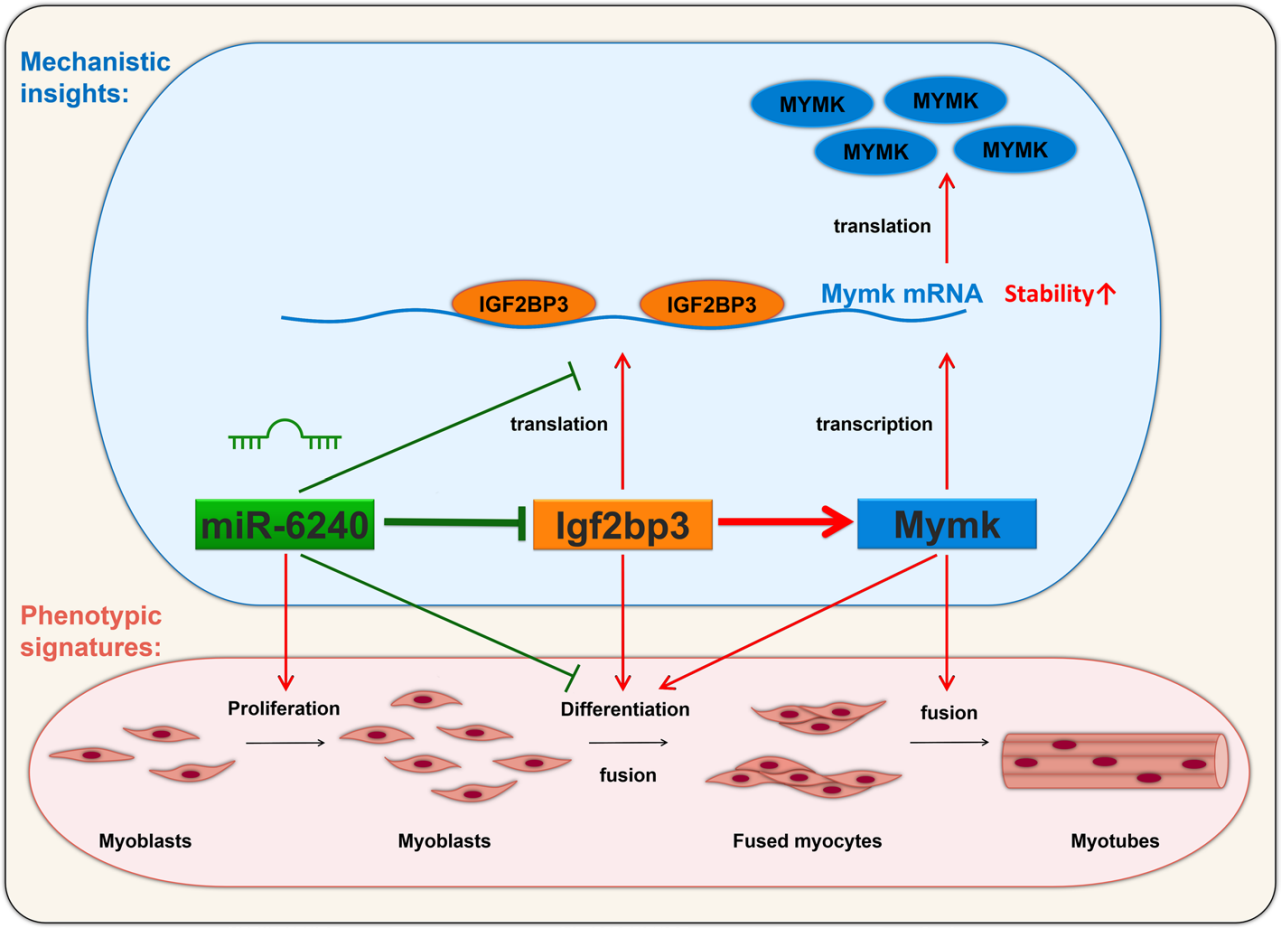

**Supplementary Information:**

The online version contains supplementary material available at 10.1186/s11658-024-00650-1.

## Background

Myogenesis is a complex physiological process that takes place during embryonic development and in adult skeletal muscle in response to damage [1–3]. A crucial stage in the myogenesis process is the fusion of myoblasts to create multinucleated myotubes [4, 5]. A wide range of regulators, including members of the myogenic regulatory factors (MRFs) family (Myf5, MyoD, MyoG, and Myf4), play key roles in modulating myoblast fusion through regulating the transcription of myogenesis genes [6]. Furthermore, the regulatory mechanism governing the fusion of myoblasts has been shown by the markers myomaker (*Mymk*) and myomixer (*Mymx*), which significantly improve the fusion capability by promoting the direct formation of multinucleated myofibers directly rather than their differentiation [7–10]. It was demonstrated that MRFs and noncoding RNAs (ncRNAs) control *Mymk* and *Mymx* primarily. Luo et al. have verified that MYOD and MYOG are bound to the *Myomaker* promoter region, thereby increasing *Myomaker* transcription in chicken myoblasts [7]. And they also discovered that miR-140-3p inhibits *Myomaker* expression and myoblast fusion by targeting the 3′ untranslated region (UTR) region of *Myomaker* [7]. Subsequent studies have also shown that miR-491 and miR-16-1 similarly regulate myofibroblast fusion through this mechanism [11–13]. To be specific, they act as post-transcriptional regulators of *Mymk*, specifically binding to the *Mymk* mRNA 3′ UTR, leading to downregulation of MYMK expression and inhibition of skeletal muscle formation. Consequently, it is helpful to learn more about the regulatory networks of *Mymk* and *Mymx* to deeply understand the myoblast fusion in myogenesis.

RNA-binding proteins (RBPs) influence the cellular transcriptome and overall state by modulating the post-transcriptional processing of RNA transcripts, such as splicing, 5′-end capping, 3′-end cleavage, and polyadenylation [14]. RBPs also regulate the translation efficiency, turnover, and subcellular localization of RNA transcripts [13–17]. Recent evidence suggests that the *N*^6^-methyladenosine (m^6^A) “reader” protein Ythdf2 could be able to bind to the m^6^A sites in STK11 mRNA. In an Ago2 system-dependent manner, Ythdf2 mediates the degradation of *STK11* mRNA, an AMPK activator, thereby driving skeletal myogenesis by inhibiting the AMPK/mTOR pathway [18]. IGF2BP, another m^6^A reader [19], is part of a conserved family of RNA-binding proteins [20]. IGF2BP3 exerts a crucial influence on myogenesis regulation through its role as an RBP. Previous studies have reported that IGF2BP3 directly binds to insulin-like growth factor 2 (IGF2), thereby regulating its translation and the expression levels of both intracellular and secreted IGF2 [20, 21]. IGF2 is a master switch governing the initiation of skeletal myogenesis [22]. At the post-transcriptional level, IGF2BP3 affects chicken primary myoblasts proliferation through regulating IGF2 expression [23]. Furthermore, the DNA methylation/SP1/IGF2BP3 axis has been linked to myogenesis, as demonstrated by our earlier research [24]. These discoveries imply that *Igf2bp3* might be involved in modulating the proliferation and differentiation of C2C12 myoblasts. However, it remains unclear what specific role *Igf2bp3* plays in myoblast fusion.

In this study, functional gain/loss experiments were carried out on *Igf2bp3* in C2C12 myoblasts in order to clarify its role and regulatory mechanisms in myogenesis. The results showed that *Igf2bp3* promotes C2C12 myoblasts fusion. Mechanistically, *Igf2bp3* expression is downregulated by miR-6240, which is achieved by targeting the *Igf2bp3* 3′ UTR. Meanwhile, miR-6240 could regulate myogenesis via *Igf2bp3*. In addition, *Igf2bp3* increases the stability of *Mymk* mRNA by binding its mRNA, favorably regulating *Mymk* expression and eventually promoting C2C12 myoblast fusion. Our study reveals *Igf2bp3* serves as a post-transcriptional regulator of myoblast fusion.

## Materials and methods

### Cell culture and transfection

The cells, including mouse C2C12 myoblasts and human embryonic kidney (HEK)-293 T cells, are preserved in our laboratory, which was obtained from the American Type Culture Collection (ATCC, USA). The* Igf2bp3* knockout (KO-*Igf2bp3*) C2C12 cell line, generated using CRISPR technology, was developed in our laboratory. Dulbecco’s modified Eagle’s medium (DMEM, Gibco, USA) was the growth medium (GM) used for cell culture [25]. It contained 1% penicillin–streptomycin (PS, Thermo Scientific, USA) and 10% fetal bovine serum (FBS, Gibco, USA). Growing to 80% density and substituting the GM with differentiation medium [DM, DMEM containing 2% horse serum (HS, Biological Industries) and 1% PS] caused C2C12 myoblasts to differentiate [26]. Lipofectamine™ 3000 (Thermo Fisher Scientific, USA) was utilized to transfect cells with siRNAs, overexpression vectors, miRNA mimics, or inhibitors for the gain and loss of function investigations. For the function analysis, the medium was switched out with either GM or DM after 6 h after transfection.

### Plasmid construction and RNA oligonucleotides

The *Igf2bp3* overexpression vector was created utilizing the NheI and KpnI restriction sites on the pcDNA3.1(+) vector. Additional file 1: Table S1 mentions the primers that were utilized in vector production. All siRNAs, miRNA mimics and inhibitors were synthesized from RiboBio (China), as listed in Additional file 1: Table S2.

### RNA isolation, reverse transcribe, and real-time quantitative polymerase chain reaction (RT-qPCR)

Total RNAs were extracted utilizing the RNAiso Plus reagent (TaKaRa, China). For reverse transcription of total RNAs into cDNAs, the HiScript III 1st Strand cDNA Synthesis kit (+gDNA wiper) (R312-01, Vazyme, China) was employed. The quantification of cDNAs was conducted using the Taq Pro Universal SYBR qPCR Master Mix (Q712-02, Vazyme, China) on an ABI QuantStudio 3^TM^ Real-Time PCR system (Applied Biosystems; USA). For miRNA reverse transcription and RT-qPCR, the miRNA 1st Strand cDNA Synthesis Kit (MR101-01, Vazyme, China) was employed. GAPDH was used as the internal normalization control for mRNA quantification, while U6 was utilized for miRNA quantification. Additional file 1: Table S3 displays the primer sequences.

### Western blot

Protein extraction from C2C12 myoblasts was performed using RIPA Lysis Buffer (Thermo Scientific, USA) supplemented with 1% phenylmethylsulfonyl fluoride (PMSF; Solarbio; China) on ice for 15 min. After centrifugation at 13,000*g* at 4 °C for 15 min, the protein fractions were collected and quantified using a BCA protein assay kit (Beyotime, China). Proteins were denatured with sodium dodecyl sulfate (SDS, Yeasen Biotechnology, China) at 100 °C for 25 min, then separated by precast 10% or 4–12% sodium dodecyl sulfate polyacrylamide gel electrophoresis (SDS–PAGE) gels (EpiZyme/Yeasen, China), and finally transferred to Hybridization Nitrocellulose Filter (NC) membranes (Merck #HATF00010, Germany), which were blocked with 5% skim milk for 2 h, incubated with specific primary antibodies for MyHC (50 ng/mL, DSHB, USA), MYMK (1:500, ABclonal, USA), PCNA (1:5000, Proteintech, USA), Cyclin A2 (1:5000, Proteintech, USA), MKI67 (1:500, Proteintech, USA), IGF2BP3 (1:5000, Proteintech, USA), histone H3 (1:1000, Abcam, England), GAPDH (1:20,000, Proteintech, USA), α-tubulin (1:5000, Proteintech, USA), and β-actin (1:10,000, Proteintech, USA) and with a secondary antibody conjugated with horseradish peroxidase (HRP; 1:5000, OriGene, China). Protein bands were visualized with enhanced ECL reagents (Yeasen, China), then analyzed by ImageJ software.

### Immunofluorescence assay

After 5 days of inducing differentiation in 12-well plates, C2C12 myoblasts were fixed with 4% paraformaldehyde for 20 min at room temperature (Beyotime, China). After fixation, the cells were permeabilized with 0.5% Triton X-100 (Sigma, USA) for 20 min, followed by blocking with 5% bovine serum albumin (BSA; Biofroxx, Germany) for 1 h. Subsequently, the cells were incubated with anti-mouse MyHC antibody (0.5 μg/mL, DSHB, USA) for 2 h before being treated with the secondary antibody (FITC/CY3, 1:500, Servicebio, China) for 1 h. Lastly, the cell nuclei were stained for 10 min in the dark using DAPI (Beyotime, China). All operations are performed at room temperature. A fluorescent inverted microscopes (OLYMPUS, BX53, Japan; SOPTOP, ICX41M, China; NIKON, A1HD25, Japan) was used to take the pictures.

### EdU assay

Cellular proliferation viability was evaluated using the BeyoClick™ EdU Cell Proliferation Kit with Alexa Fluor 555 (Beyotime, Shanghai, China). Cells were treated with a medium containing 10 μM EdU at 37 °C and 5% CO_2_ for 2 h following transfection; subsequently, the cells were fixed with 4% paraformaldehyde at room temperature for 15 min. After three 5-min washes with PBS, the cells were permeabilized for 10 min using 0.5% Triton X-100. Next, the cells were incubated with Click Reaction Reagent for 30 min at room temperature in the dark, following the manufacturer’s protocols. Finally, nuclei were stained with DAPI dye for 10 min. Fluorescence microscopy was used to take the images. The ratio of EdU-positive cells was determined by dividing the number of EdU-positive cells by the number of DAPI-stained cells, and then multiplying by 100%.

### CCK-8 assay

C2C12 myoblasts were seeded into a 96-well plate, and their growth was assessed by treating with CCK-8 reagent (10 μL/well, Beyotime, Shanghai, China) at 0 h, 24 h, 48 h, and 72 h post-transfection, followed by a 40-min incubation at 37 °C with 5% CO_2_. Subsequently, the absorbance at 450 nm was measured using a microplate reader.

### Cloning and dual-luciferase reporter assay

The *Igf2bp3* mRNA 3′ UTR fragments, which included the binding site of miR-6240, were generated using specific primers, and the amplified products were then inserted into the pmirGLO vectors using restriction enzymes NheI and XhoI (Thermo Fisher Scientific, USA), downstream of the firefly luciferase open reading frame (ORF). We refer to the wild-type (WT) vector as pmirGLO-*Igf2bp3* 3′ UTR-WT. The mutant (MT) pmirGLO-*Igf2bp3* 3′ UTR-MT was created by altering complementary to the *Igf2bp3* seed region using mutagenic primers. Two micrograms of either WT or MT plasmids were transfected into cells in 12-well plates for reporter experiments using Lipofectamine™ 3000. After 24 h, the cells were lysed, and relative luciferase activities were assessed using a Dual-Luciferase Reporter Assay kit (Promega, Wisconsin, USA) on a GloMax™ 20/20 Luminometer (Promega, Wisconsin, USA) following the manufacturer’s instructions.

### RNA immunoprecipitation qPCR (RIP-qPCR) assay

Two T75 cell culture flasks were seeded with C2C12 myoblasts, which were harvested upon transfection and growing to confluence. The RNA Immunoprecipitation (RIP) Kit (BerSinBio #Bes5101, Guangzhou, China) was then used for RIP experiments following compliance with the manufacturer’s instructions. *Igf2bp3* and miR-6240 were immunoprecipitated using an argonaute 2 antibody (anti-AGO2, Abcam ab186733, Cambridge, UK), while *Mymk* was immunoprecipitated using an IGF2BP3 polyclonal antibody (Proteintech Group #14,642–1-AP, Chicago, USA). Subsequently, RNA was extracted from the immunoprecipitated materials, and RT-qPCR analysis was performed.

### RNA stability analyses

Following transfection with *Igf2bp3* siRNA or a negative control, C2C12 myoblasts were treated with actinomycin D (Act-D, 5 µg/mL). Total RNA was then extracted at 0, 4, and 8 h after Act-D treatment, and the relative levels of *Mymk* mRNA were assessed by RT-qPCR [27]. The half-life (*t*_1/2_) of mRNAs was determined as the time required to reach 50% of the initial mRNA abundance at time 0 before the addition of Act-D [2].

### Statistical analysis

The fusion index, representing the proportion of nuclei in fused myotubes with two or more nuclei compared to the total number of nuclei, was calculated and evaluated using ImageJ software [28]. In this work, protein band grey value analysis and EdU cell counting were also conducted using ImageJ program. All statistical analysis and graph generation were conducted by Graphpad Prism 8.0.2 (California, USA). For every set of data, the mean ± standard error of mean (SEM) is presented. Statistical analysis employed the student’s *t*-test to compare outcomes. *P* values below 0.05 were considered statistically significant.

## Result

### Expression and subcellular localization of *Igf2bp3* in myogenesis

**Fig. 1:**
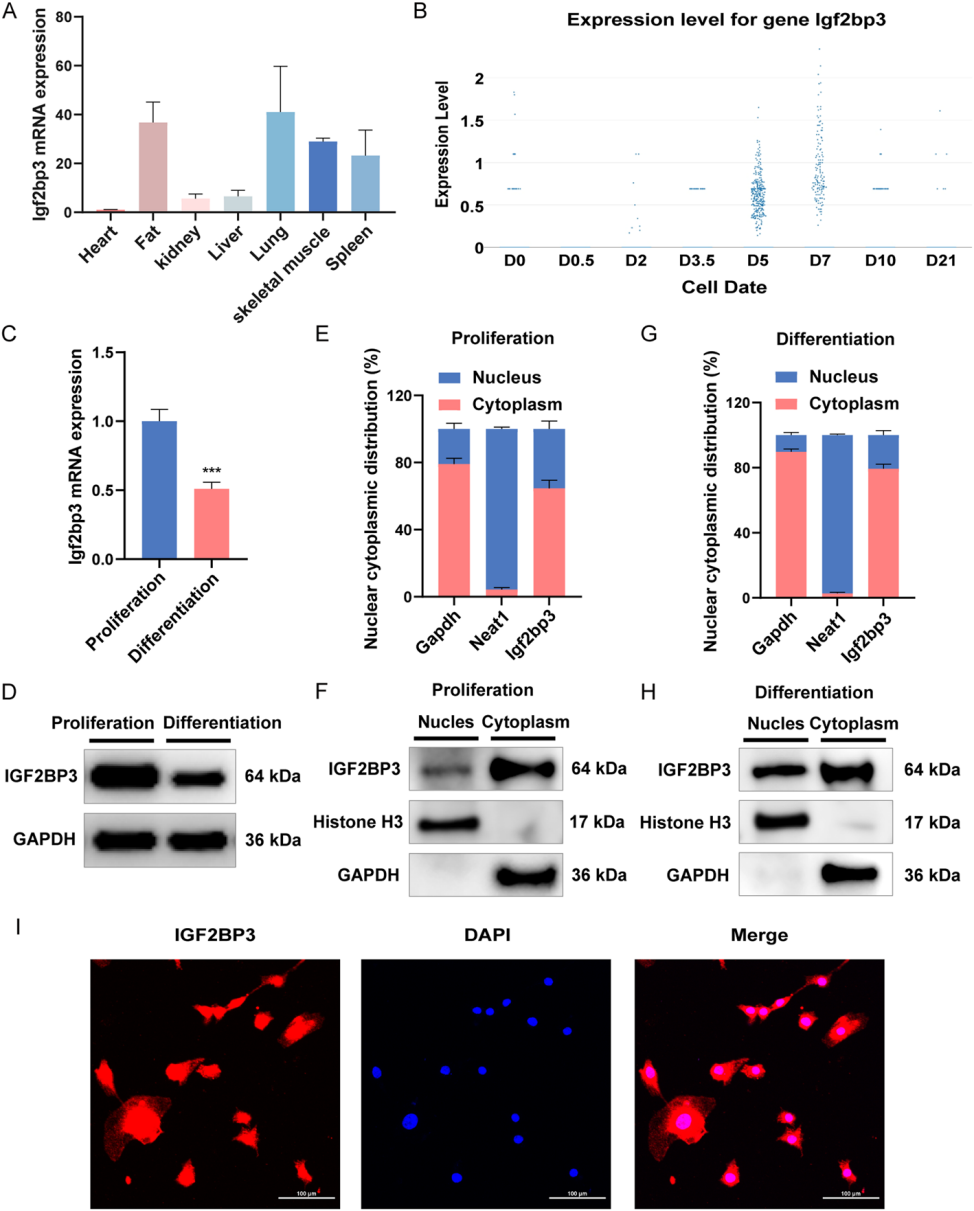
The expression and cellular localization of *Igf2bp3*. **A** The *Igf2bp3* expression pattern in various tissues of 12-week-old mice. **B** The expression of *Igf2bp3* during regeneration is based on the SCSMRD database. **C**, **D** RT-qPCR and western blot to quantify the *Igf2bp3* mRNA (**C**) and protein (**D**) levels in C2C12 cells that were differentiating and proliferating, respectively. **E**, **G** The distribution of *Igf2bp3* in the nucleus (blue) and cytoplasm (red) was detected by RT-qPCR after purification of nuclear and cytoplasmic RNAs from proliferating (E) and differentiating (G) C2C12 myoblasts. **F**, **H** Western blot to quantify IGF2BP3 expression in the nucleus and cytoplasm of proliferating (**F**) and differentiating (**H**) C2C12 myoblasts. **I** IGF2BP3 subcellular location in C2C12 myoblasts was identified using immunofluorescence. DAPI-stained nuclei are shown in blue, while IGF2BP3 staining is shown in red. Scale bars, 100 μm. For normalization, Neat1, GAPDH, and histone H3 were employed. *N* = 3 in each group. Data are represented as mean ± SEM. ***P < 0.001 (Student’s *t*-test)

The expression of* Igf2bp3* was initially investigated in various tissues of adult C57BL/6 mice. RT-qPCR results showed that *Igf2bp3* was abundantly expressed in the adipose and skeletal muscle (Fig. 1A). Next, using SCSMRD, a single-cell database for skeletal muscle regeneration [29], we found the expression of *Igf2bp3* was upregulated during regeneration, and reached the peak at day 5 and 7 post-injury (Fig. 1B). In addition, the expression changes of *Igf2bp3*, *Pax7*, *Myod1*, and *Myog* during CTX-induced mouse skeletal muscle regeneration were analyzed along with public RNA-seq data [30]. The results are shown in Additional file 1: Fig. S1B.* Igf2bp3* was found to be more highly expressed during the proliferation stage of myogenesis compared to differentiation stage, as demonstrated by both RT-qPCR and western blot (Fig. 1C, D). We further detected the expression changes of *Igf2bp3* during C2C12 myoblast differentiation, and the results are shown in Additional file 1: Fig. S1A. These findings revealed a potential role for *Igf2bp3* in myogenesis and skeletal muscle regeneration.

We next investigated the localization of *Igf2bp3*. *Igf2bp3* exhibited a dual presence in both cellular compartments of C2C12 myoblasts, with a noticeably higher concentration within the cytoplasmic region (Fig. 1E–H). Interestingly, this difference in expression between the two compartments remained relatively consistent throughout the proliferation and differentiation phases. Furthermore, immunofluorescence experiments confirmed that IGF2BP3 was largely located in the cytoplasm of proliferating C2C12 myoblasts (Fig. 1I).

### *Igf2bp3* positively regulates the differentiation and fusion of myoblasts

According to our earlier research, C2C12 myoblasts were greatly delayed in differentiating and induced to proliferate when *Igf2bp3* was knocked down [24]. To confirm the functions of *Igf2bp3* in myogenesis, we synthesized two independent siRNAs (si1-*Igf2bp3* and si2-*Igf2bp3*) to knockdown *Igf2bp3* and generated a stable *Igf2bp3* knockout (KO-*Igf2bp3*) C2C12 cell line using CRISPR technology. The results confirmed that knockdown and knockout of *Igf2bp3* repressed myoblast differentiation and promoted myogenic proliferation (Additional file 1: Figs. S2A–C, S3A–C, and S6A–H). Meanwhile, we used recombinant plasmid pcDNA3.1(+) to overexpress *Igf2bp3* and found that overexpression of *Igf2bp3* significantly enhanced the expression of differentiation markers in C2C12 myoblasts (Additional file 1: Fig. S2D, E). In addition, the analysis of cell differentiation involved the use of an antibody against the myosin heavy chain (MyHC) to determine the myogenic differentiation index [31]. Immunofluorescence staining revealed that the overexpression of *Igf2bp3* led to an augmentation in MyHC immunostaining and differentiation index (Additional file 1: Fig. S2F). Correspondingly, *Igf2bp3 *overexpression confirmed the impact of *Igf2bp3* on C2C12 myoblast proliferation (Additional file 1: Fig. S6I-L). In conclusion, these findings supported the notion that *Igf2bp3* enhances the myogenic differentiation while suppressing myoblasts proliferation in C2C12 cells.

**Fig. 2:**
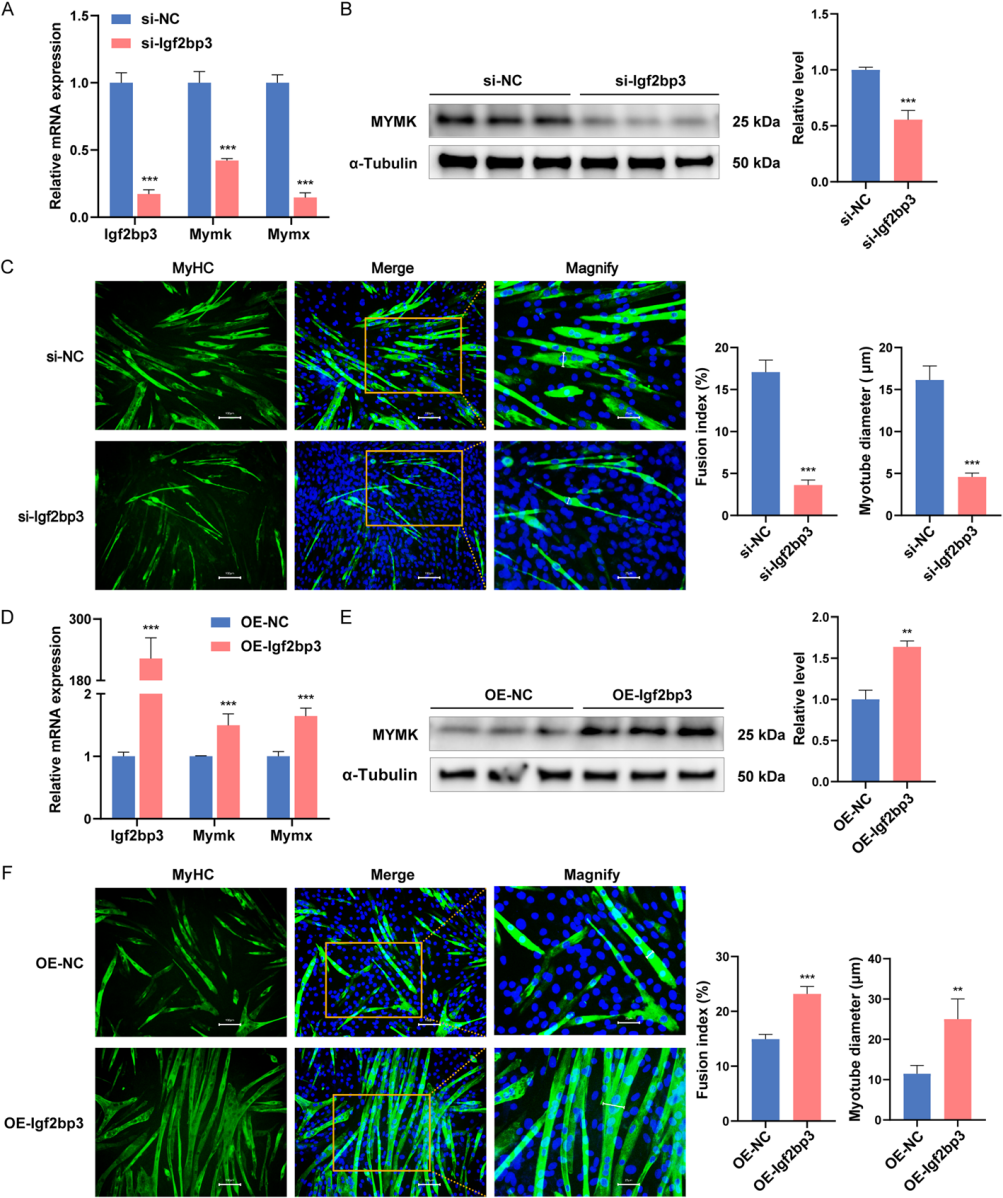
The function of *Igf2bp3* on myoblast fusion. **A**, **B** mRNA (**A**) and protein (**B**) levels of *Igf2bp3* and fusion markers were measured by RT-qPCR and western blot following transfection with *Igf2bp3* siRNA or a negative control. **C** Immunofluorescence detection of MyHC (green) in C2C12 myoblasts transfected with *Igf2bp3* siRNA or a negative control. To detect cell nuclei, DAPI (blue) was utilized. Measurements were made of the fusion index and the myotube diameter. **D**, **E** The expression levels of *Igf2bp3* and fusion markers were measured by RT-qPCR (**D**) and western blot (**E**) after* Igf2bp3* overexpression. **F** Immunofluorescence analysis of C2C12 myoblasts overexpressing *Igf2bp3*, stained with MyHC (green). The cell nuclei were visible with DAPI (blue). For **C** and **F**, micrographs of MyHC and merge were taken using 200× magnification (scale bar, 100 µm) and magnified the partial part of the merge image was magnified by 4× (scale bar, 25 µm). *Gapdh* or α-tubulin was utilized as an internal control. *N* = 3 in each group. Data are represented as mean ± SEM. **P < 0.01; ***P < 0.001 (Student’s *t*-test)

We next investigated the effects of *Igf2bp3* on myoblast fusion. C2C12 myoblasts were transfected with two *Igf2bp3* siRNAs or the constructed pcDNA3.1(+)-*Igf2bp3* plasmid. RT-qPCR and western blotting analysis suggested that the knocking down Igf2bp3 markedly downregulated the mRNA expression of *Mymk* and *Mymx* (Fig. 2A and Additional file 1: Fig. S3D) and the protein expression of MYMK (Fig. 2B and Additional file 1: Fig. S3E). Overexpression of *Igf2bp3* significantly increased their expression (Fig. 2D, E). Meanwhile, the knockdown of *Igf2bp3* in C2C12 myoblasts results in fewer and smaller myotubes (Fig. 2C and Additional file 1: Fig. S3F). In contrast, overexpression of *Igf2bp3* promoted myoblast fusion and generated larger myotubes with more nuclei when compared with control myoblasts (Fig. 2F). Taken together, these results reveal that *Igf2bp3* inhibits myoblasts proliferation, promotes myogenic differentiation and fusion.

In addition, we also explored the functions of *Igf2bp3* in myogenesis using mouse primary myoblasts. The results showed that knockdown of *Igf2bp3* by two siRNAs both significantly decreased the mRNA and protein expression of *MyHC* compared to the control groups (Additional file 1: Fig. S4A, B). Meanwhile, immunofluorescence showed that primary myoblast differentiation is inhibited following *Igf2bp3* knocking down (Additional file 1: Fig. S4C). Furthermore, the downregulation of *Igf2bp3* expression decreased the mRNA and protein expression of *Mymk* and inhibited myotube formation (Additional file 1: Fig. S5A-C). In contrast, overexpression of *Igf2bp3* promoted differentiation and fusion of primary myoblasts (Additional file 1: Figs. S4D-F and S5D-F).

### miR-6240 directly targets 3′ UTR of *Igf2bp3*

**Fig. 3:**
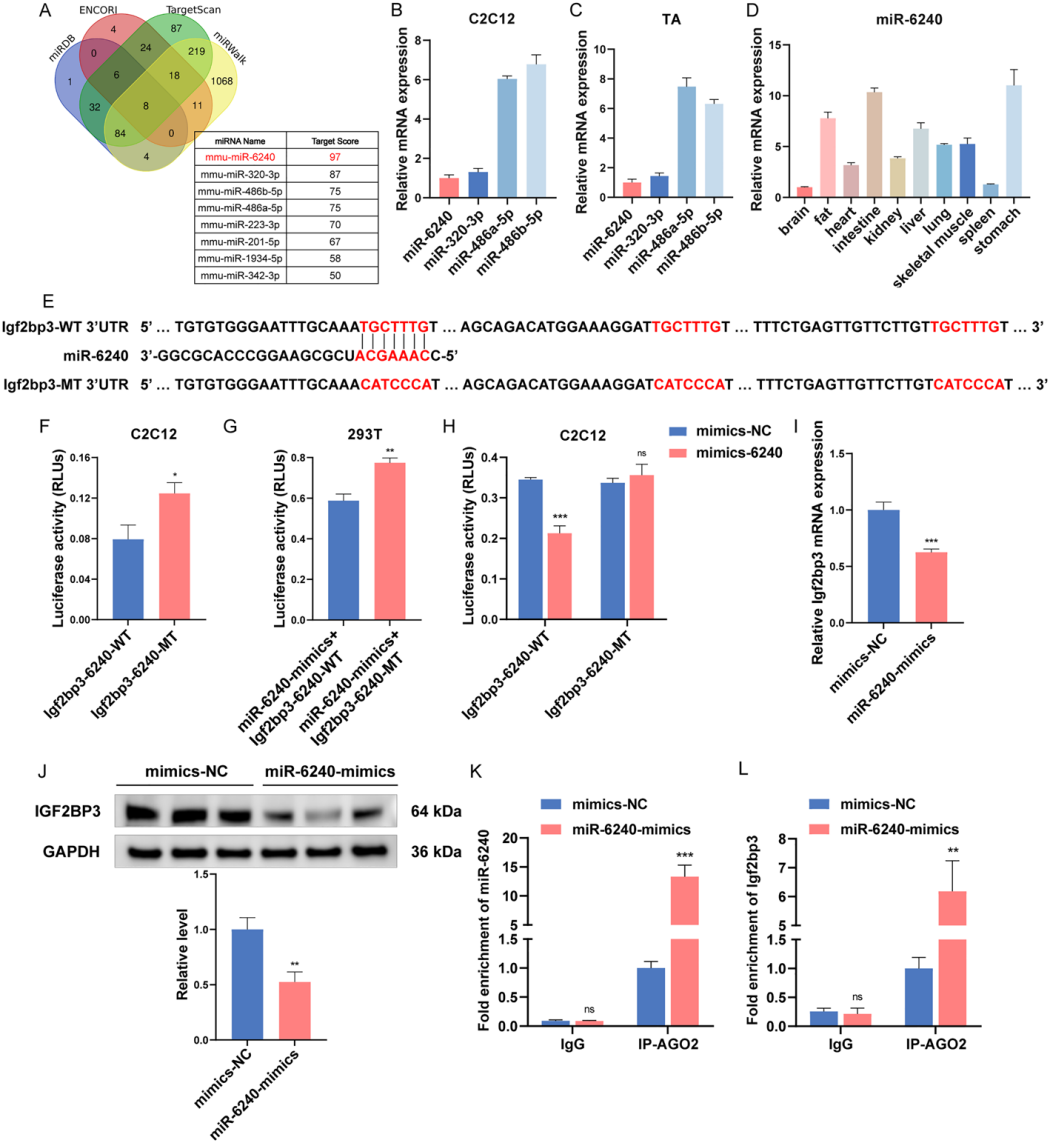
*Igf2bp3* is a target for miR-6240. **A** Potential miRNAs targeting *Igf2bp3*. **B**, **C** RT-qPCR showing the expression of candidate miRNAs in C2C12 (**B**) and mouse TA muscle (**C**). **D** RT-qPCR showing the expression pattern of miR-6240 in different mouse tissues. **E** Diagram showing the WT/MT reporter constructs that are utilized to pinpoint the *Igf2bp3* 3' UTR’s miR-6240 target areas. **F** The relative ratios of firefly luciferase (FL) to renilla luciferase (RL) were ascertained 24 h following the transfection of the plasmids into C2C12 myoblasts. **G** The plasmids shown were co-transfected with miR-6240 mimics into HEK-293T cells; they were cultured for 24 h, whereupon the relative FL/RL ratios were calculated. **H** The plasmids shown were co-transfected with either control miRNA or miR-6240 mimics into C2C12 myoblasts; they were cultured for 24 h, and then the relative FL/RL ratios were determined. **I**, **J** The expression of both *Igf2bp3* mRNA (**I**) and protein (**J**) were downregulated by miR-6240 mimics. **K**, **L** The RIP experiment's enrichment of miR-6240 (**K**) and *Igf2bp3* (**L**). GAPDH or U6 was utilized as an internal control. *N* = 3 in each group. Data are represented as mean ± SEM. *P < 0.05; **P < 0.01; ***P < 0.001; ns, not significant (Student’s *t*-test)

miRNAs exert their functions mainly by binding to the mRNA 3′ UTR of target genes and inhibiting their expression [32]. To explore the regulatory mechanism of *Igf2bp3* affecting myogenesis, we employed four programs (miRDB, ENCORI, TargetScan, and miRWalk) to predict miRNAs that could potentially target *Igf2bp3*. According to Fig. 3A, eight miRNAs, including myogenic miR-486 [33, 34], were predicted by all the programs to bind the 3′ UTR of *Igf2bp3*. The miRNA with the greatest prediction score was miR-6240 (Fig. 3A). Next, we analyzed the expression of four miRNAs with highest scores in C2C12 myoblasts and mouse tibialis anterior (TA) muscle tissues, and found that miR-486 was abundantly expressed, while the expressions of miR-6240 and miR-320-3p were similar to each other (Fig. 3B, C). Then, we investigated the tissue profile of miR-6240 and noted its abundant expression level in skeletal muscle (Fig. 3D). Furthermore, the expression changes of miR-6240 during C2C12 myoblast differentiation and during CTX-induced regeneration in TA muscles were detected (Additional file 1: Fig. S7A, D). Interesting, miR-6240 expression is significantly higher at the proliferation phase compared to the differentiation phase (DM 48, 72, 96, and 120 h), which are similar to those observed in *Igf2bp3*. Similarly, miR-6240 is mostly localized in the cytoplasm, both in the proliferating and differentiated C2C12 myoblast (Additional file 1: Fig. S7B, C). The purpose of our next focus was to validate whether miR-6240 could target *Igf2bp3* directly.

WT or MT *Igf2bp3* 3′ UTR sequences were included in the dual-luciferase reporters (Fig. 3E), and were transfected into C2C12 myoblasts. The luciferase activity of the MT vector was significantly higher compared with that of the WT group (Fig. 3F). Additionally, we co-transfected WT or MT *Igf2bp3* 3′ UTR reporters into HEK-293T together with miR-6240 mimics. Comparable outcomes to those observed in C2C12 myoblasts were discovered (Fig. 3G). We further co-transfected them into C2C12 myoblasts. The findings demonstrated that, in myoblast co-transfected with the WT reporters and miR-6240 mimics, luciferase activity was reduced in comparison to control, whereas no alterations were seen in cells co-transfected with the MT reporters (Fig. 3H). In addition, overexpression of miR-6240 resulted in downregulation of *Igf2bp3* expression at both the mRNA and protein levels (Fig. 3I, J). However, the opposite effect was observed when miR-6240 was inhibited (Additional file 1: Fig. S10A, B). Furthermore, RIP assay revealed a significant enrichment of both miR-6240 and *Igf2bp3* within anti-AGO2 pellets. This observation strongly suggested that the binding interaction between miR-6240 and the *Igf2bp3* 3' UTR led to the co-precipitation of these elements, indicating a direct interaction between miR-6240 and *Igf2bp3* (Fig. 3K, L). In conclusion, these findings indicate that miR-6240 potentially targets the 3' UTR of* Igf2bp3*.

### miR-6240 regulates myogenesis by targeting *Igf2bp3*

The role of miRNA-6240 in myogenesis was then investigated. We discovered that transfection of C2C12 myoblasts with miR-6240 mimics significantly enhanced the expression of proliferative markers at both the mRNA and protein levels (Additional file 1: Fig. S8A, B); the opposite was observed after inhibition of miR-6240 (Additional file 1: Fig. S10C, D). Additionally, the EdU assay revealed a significant increase in the number of EdU-positive cells in the miR-6240 mimics-treated group compared with that in the negative control (NC) counterparts (Additional file 1: Fig. S8C). However, the opposite effect was observed in the group treated with miR-6240 inhibitors (Additional file 1: Fig. S10E). The CCK-8 and flow cytometry assays further confirmed that the proliferation activity was increased significantly in myoblasts transfected with miR-6240 mimics (Additional file 1: Fig. S8D, E). Conversely, the growth was delayed when miR-6240 was inhibited in myoblasts (Additional file 1: Fig. S10F). Meanwhile, on day 3 of differentiation, overexpression of miR-6240 markedly inhibited the expression of differentiation markers in myoblasts (Additional file 1: Fig. S8F, G), while their expression was increased with miR-6240 inhibition (Additional file 1: Fig. S10G, H). Furthermore, the immunofluorescence assay of MyHC indicated that overexpression of miR-6240 suppressed myotube formation, resulting in reductions in both the size and number of myotubes (Additional file 1: Fig. S8H), but the result was reversed when miR-6240 was inhibited (Additional file 1: Fig. S10I). These results revealed that miR-6240 stimulated myoblast proliferation and inhibited myoblast differentiation.

**Fig. 4:**
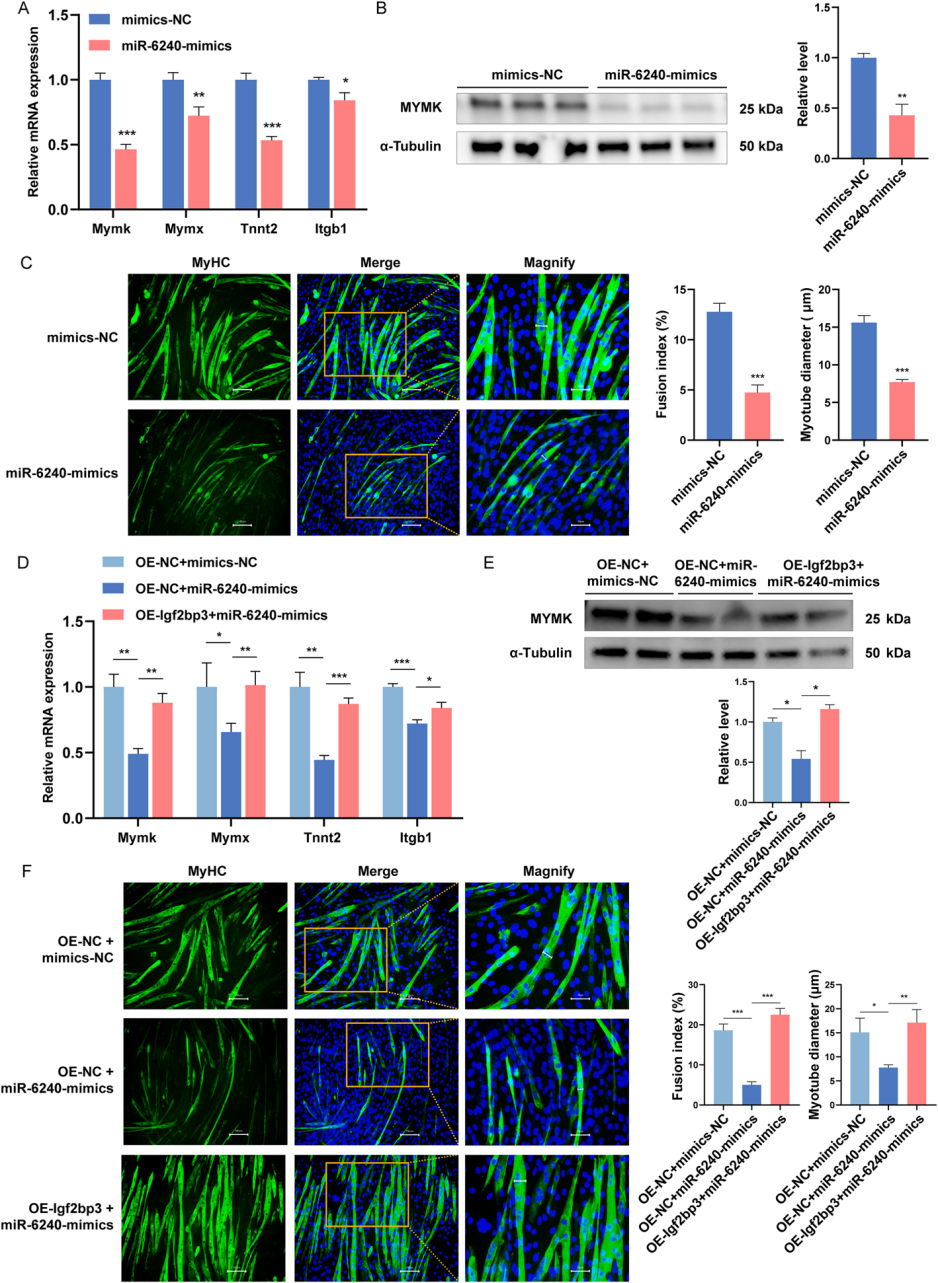
miR-6240 inhibits myoblast fusion through regulating *Igf2bp3*. **A**, **B** After transfection with miRNA-6240 mimics or a negative control, the levels of fusion markers were detected by RT-qPCR (**A**) and western blot (**B**). **C** C2C12 myoblasts were transfected with miRNA-6240 mimics or a negative control; their fusion was tracked by measuring the diameter of the myotube, the fusion index, and the MyHC (green) levels using immunofluorescence. With the use of DAPI (blue), cell nuclei were seen. **D**, **E** After co-transfection with miR-6240 mimics and the pcDNA3.1(+) vector or a negative control, the levels of fusion markers were detected by RT-qPCR (**D**) and western blot (**E**). **F** MyHC-stained (green) C2C12 myoblasts that were induced to fuse for five days following transfection were examined using immunofluorescence. DAPI (blue) was used to visualize the cell nuclei. For **C** and **F**, micrographs of MyHC and merge were taken using 200× magnification (scale bar, 100 µm) and magnifying the partial part of the merge image by 4× (scale bar, 25 µm). Quantification was performed on the fusion index and myotube diameter. *Gapdh* and α-tubulin were used for standardization. *N* = 3 in each group. Data are represented as mean ± SEM. *P < 0.05; **P < 0.01; ***P < 0.001 (Student’s *t*-test)

We also investigated whether miR-6240 affected myoblast fusion. The results showed that overexpression of miR-6240 by miRNA mimics could downregulate the expression of fusion markers (Fig. 4A, B) in C2C12 myoblasts. In contrast, the expression of fusion markers increased when miR-6240 was inhibited (Additional file 1: Fig. S11A, B). We next examined myoblasts fusion using the immunofluorescence assay and found that overexpressing miR-6240 appears to attenuate myoblasts fusion and myotubes formation by 5 days of differentiation (Fig. 4C). In contrast, inhibition of miR-6240 resulted in a significant increase in myotube diameter and fusion index (Additional file 1: Fig. S11C). The analysis of myotube formation revealed that overexpression of miR-6240 decreased the fusion index approximately from 14% to 5% after 5 days of differentiation, along with a significant reduction in the mean diameter of myotubes (Fig. 4C). Taken together, these results indicate that miR-6240 promotes myoblasts proliferation and inhibits myogenic differentiation and fusion, which was opposite to the function of *Igf2bp3*.

To determine whether miR-6240 regulates myogenesis by targeting *Igf2bp3*, we performed the co-transfection assay of pcDNA3.1(+)-*Igf2bp3* or pcDNA3.1(+) vectors with miR-6240 mimics or mimics-NC. The results showed that co-transfection of miR-6240 mimics and pcDNA3.1(+) vector into myoblasts upregulated the expression of proliferation genes; however, co-transfection of miR-6240 mimics and pcDNA3.1(+)-*Igf2bp3* vector brought them down to the initial levels (Additional file 1: Fig. S9A, B). EdU and CCK8 analyses further confirmed that *Igf2bp3* overexpression counteracted the promotional effect of miR-6240 on the myoblasts proliferation (Additional file 1: Fig. S9C, D). Moreover, the number of cells arrested in the G0/G1 phase decreased, while the cell number in the S phase increased due to miR-6240 overexpression; nevertheless, this effect was countered by simultaneous overexpression of *Igf2bp3* (Additional file 1: Fig. S9E). The inhibition of miR-6240 on myogenic differentiation was rescued by *Igf2bp3* overexpression (Additional file 1: Fig. S9F-H). Furthermore, *Igf2bp3* overexpression can rescue the effects of miR-6240 on the expression of myoblast fusion markers (Fig. 4D, E). The immunofluorescence assay demonstrated that the inhibition of myotube formation induced by transfection with miR-6240 mimics was rescued by the overexpression of *Igf2bp3*, thereby partially restoring myogenesis (Fig. 4F). Overall, these results confirm that the promotion of proliferation and reduction of differentiation and fusion elicited by miR-6240 is mediated by targeting *Igf2bp3*.

### *Igf2bp3* enhances the mRNA stability of *Mymk*

**Fig. 5:**
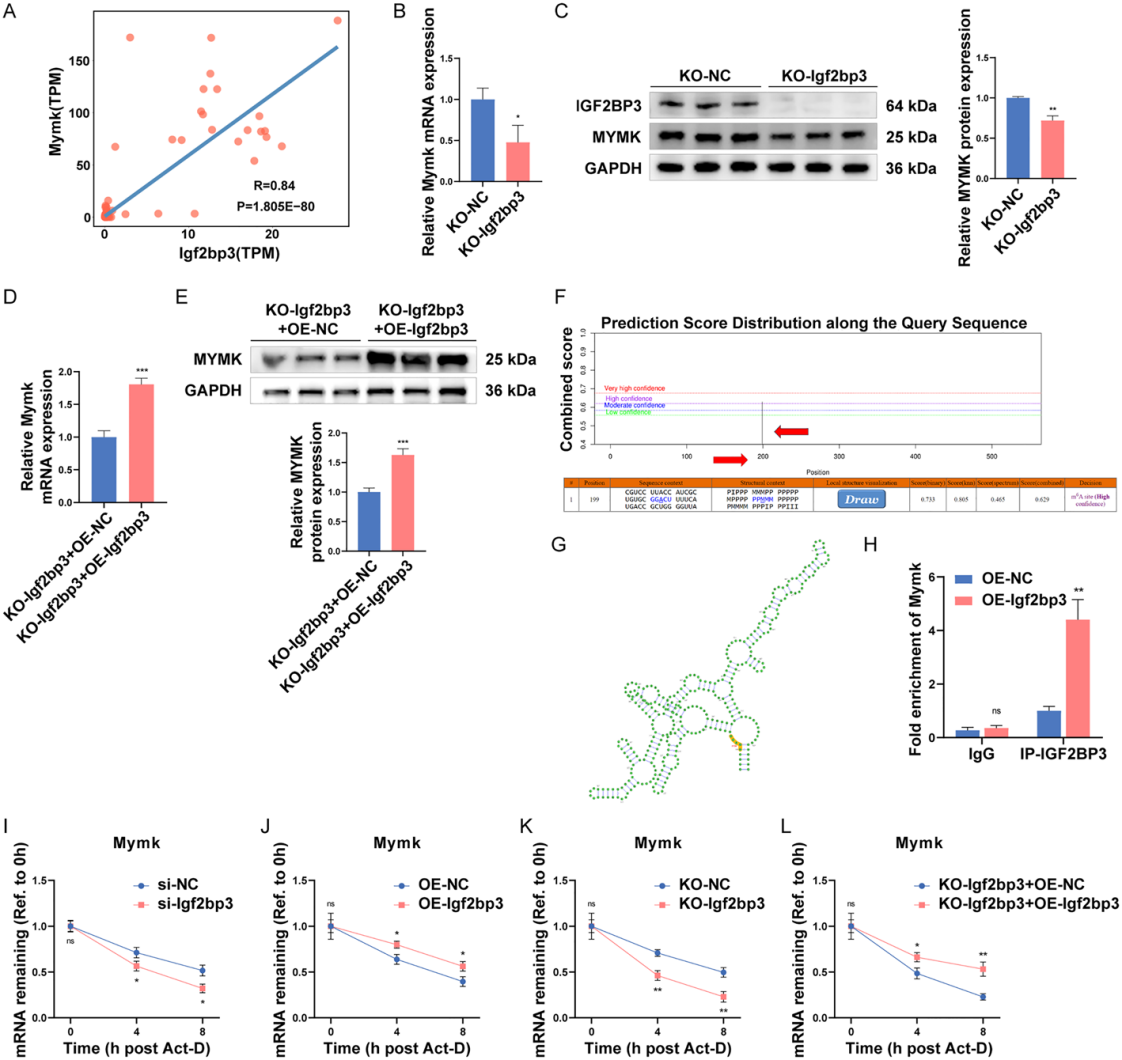
*Igf2bp3* regulates the expression of *Mymk* through affecting mRNA stability. **A** The expression correlation between *Igf2bp3* and *Mymk* in skeletal muscle. **B**, **C** The *Mymk* mRNA (**B**) and protein (**C**) expressions were assessed in the KO-*Igf2bp3* C2C12 cell line on day 5 of differentiation. **D**, **E** The KO *Igf2bp3*-C2C12 cell line was transfected with pcDNA3.1(+)-*Igf2bp3* or a negative control plasmid, followed by assessment of *Mymk* mRNA (**D**) and protein (**E**) levels. **F**, **G** Potential m^6^A modification site (**F**) and secondary structure (**G**) of *Mymk* predicted by the SRAMP website. **H** The enrichment of *Mymk* in the RIP experiment. **I** The stability of the *Mymk* mRNA was impaired by the knockdown of *Igf2bp3*. **J** The stability of the *Mymk* mRNA was enhanced by the overexpression of *Igf2bp3*. **K** The stability of the *Mymk* mRNA was significantly reduced in the KO-*Igf2bp3* C2C12 cell line. **L** The KO-*Igf2bp3*-C2C12 cell was transfected with pcDNA3.1(+)-*Igf2bp3* or a negative control plasmid, followed by assessment of *Mymk* mRNA stability. GAPDH was utilized as an internal control. *N* = 3 in each group. Data are represented as mean ± SEM. *P < 0.05; **P < 0.01; ***P < 0.001; ns, not significant (Student’s *t*-test)

Considering the effect of *Igf2bp3* on myoblast fusion, we speculated that *Igf2bp3* could directly target the fusion marker genes and thus participate in the cell fusion process. A strong correlation was observed between the expressions of *Igf2bp3* and *Mymk* (Pearson’s *r* = 0.84, *P* = 1.81 × 10^−80^) according to our skeletal muscle mutiomic database (http://skmatlas.cn/) (Fig. 5A). We examined the expression of *Mymk* in the established KO-Igf2bp3 C2C12 cell line and found that it was markedly decreased (Fig. 5B, C). To further validate the effect of *Igf2bp3* on *Mymk* gene, the pcDNA3.1(+)-*Igf2bp3* plasmid was transfected into the KO-*Igf2bp3* C2C12 cell line, and the results indicated that the expressions of *Mymk* at mRNA and protein levels were restored by rescuing *Igf2bp3* (Fig. 5D, E). Next, we used RIP experiments to determine if *Igf2bp3* directly interacted with *Mymk* to modulate *Mymk* expression. The RIP–qPCR primers were designed according to potential m^6^A modification site of *Mymk*, which was predicted by SRAMP [35] (Fig. 5F, Additional file 1: Table. S3). And the secondary structure of *Mymk* and its potential m^6^A modification site is shown in the Fig. 5G. The RIP assay revealed a significant enrichment of *Mymk* in the *Igf2bp3* overexpression group (Fig. 5H). These findings imply that *Mymk* expression is positively regulated by *Igf2bp3*, which directly targets the *Mymk*.

To examine whether *Igf2bp3* regulates *Mymk* expression through a post-transcription mechanism, we assessed the effect of *Igf2bp3* on the mRNA stability of *Mymk*. Following *Igf2bp3* siRNA or pcDNA3.1(+)-*Igf2bp3* plasmid transfection, we administered Act-D (5 µg/mL) to the cultured C2C12 myoblasts in order to disrupt the transcription process [27]. At 0, 4, and 8 h after Act-D administration, we collected total RNA from C2C12 myoblasts and observed that the stability of *Mymk* mRNA was significantly decreased in the *Igf2bp3* siRNA group, but markedly increased in the *Igf2bp3* overexpressed group, compared with their respective controls (Fig. 5I, J). Besides, these results were further confirmed by the dramatically decreased stability of *Mymk* mRNA in the KO-*Igf2bp3* cell line (Fig. 5K). Moreover, overexpressing *Igf2bp3* in the KO-*Igf2bp3* cell line prevented the decrease of *Mymk* mRNA stability (Fig. 5L). Collectively, these results demonstrate that *Igf2bp3* promotes myoblasts fusion by enhancing the stability of *Mymk* mRNA.

## Discussion

The development and maintenance of skeletal muscle are crucial for the health of both animals and humans [34, 36, 37]. Moreover, comprehending the regulatory mechanisms underlying skeletal muscle development necessitates the identification of key factors that influence myogenesis [38]. Here, we report a novel regulator, *Igf2bp3*, which promotes myoblast fusion by targeting *Mymk* at the post-transcriptional level.

IGF2BP3 belongs to the IGF2BP family, which plays a direct role in regulating IGF2 mRNA translation and stability [39, 40]. IGF2 serves as a pivotal regulator of skeletal muscle development [41] and an autocrine signal in the growth process of myoblasts [42]. Lin et al. found that the expression of IGF2BP3 in the skeletal muscle of normal chickens was substantially higher compared with that of dwarf chicks (all chickens were 7 weeks old) [23]. In addition, IGF2BP3 has been shown to significantly enhance proliferation and differentiation, accelerating the generation of myotubes in chicken skeletal muscle satellite cells [43]. However, our recent study suggested that *Igf2bp3* promotes differentiation while inhibiting proliferation of C2C12 myoblasts, and this regulation is mediated by DNA methylation, which modulates SP1 binding in myogenesis [24]. In this study, by knocking down *Igf2bp3* using a different siRNA, knocking out *Igf2bp3* using a CRISPR-generated stable knockout C2C12 cell line, and constructing an *Igf2bp3* overexpression vector, we confirmed that *Igf2bp3* inhibits myoblast proliferation but promotes differentiation. The varying functions of a gene across different systems or species are commonly observed in other studies. For instance, HDAC4 has been reported to promote proliferation and inhibit myogenic differentiation in mouse C2C12 myoblasts [37, 44]. However, Zhao et al. found that HDAC4 knockdown inhibits both proliferation and differentiation in chicken SMSCs [45]. Similarly, Wang et al. demonstrated that miR-194-5p mimic transfection increased the expression of myoblast differentiation markers (Myf5 and MyoG) in C2C12 cells, suggesting miR-194-5p promotes myoblast differentiation [46]. However, Shi et al. later showed that miR-194-5p negatively regulates both proliferation and differentiation in rabbit SMSCs by targeting Mef2c [47]. These discrepancies may arise from factors such as cell type, species, or culture conditions.

Myogenesis comprises precursor cell recruitment, myoblast differentiation, and mononuclear myoblast fusion, etc. During the development of embryonic skeletal muscle, PAX7^+^ precursor cells (muscle-derived stem cells) are recruited as myoblasts. They subsequently undergo proliferation and differentiation, eventually fusing with each other or with existing muscle fibers to generate multinucleated and mature myotubes [48]. Similarly, when skeletal muscle is injured by exogenous, the dormant muscle stem cells [38] located at the base of muscle fibers become activated. They undergo proliferation and differentiation, ultimately fusing together to form new myotubes, thus facilitating the repair of muscle wounds [49]. Myoblast fusion is thus an essential stage in myogenesis. The function and regulation of* Igf2bp3* in myoblast fusion and myotube formation are still largely unclear to date. To fill the gap in this field, this study employed functional gain/loss experiments on *Igf2bp3* to determine its effect on myoblast fusion. It is noted that significant overexpression of *Igf2bp3* at the mRNA level moderately increased the expression of *Mymk* and *Mymx*. This discrepancy may result from post-transcriptional regulation or saturation effects, where additional *Igf2bp3* does not translate proportionally into functional protein. Furthermore, this highlights the complexity of regulatory networks in myogenesis, where other limiting factors may modulate the downstream effects of *Igf2bp3*. In conclusion, our results demonstrated that *Igf2bp3* can efficiently promote fusion.

It is well-established that miRNAs typically bind to the 3′ UTRs of mRNAs to suppress their translation [50–52]. miRNAs have been implicated in regulating myoblast fusion and influencing skeletal muscle development [53–55]. For instance, miR-151 and miR-5100-transfected bone marrow stromal cells have been shown to increase myoblast fusion in an IGFBP2-dependent manner [56]; miR-205 targets the *MYMK* gene to control the fusion of porcine myoblasts [57], and miR-140-3p inhibits *Mymk* expression and chicken myoblast fusion by targeting the 3′ UTR region of *Mymk* [7]. Eight miRNAs, including miR-486, which is known as a myogenic miRNA [33] and an essential component of a myogenesis regulatory network that includes *Pax7*, *MyoD*, myostatin, and NF-κB [58–60], were predicted in this study as potentially targeting the 3′ UTR of *Igf2bp3*. Considering the established role of miR-486 in skeletal muscle biology [33], we deemed that the other miRNAs among the eight predicted miRNAs may also be functionally relevant to skeletal muscle development. Among these miRNAs, miR-6240 exhibited the most noteworthy prediction score, indicating a strong likelihood of its interaction with *Igf2bp3*.

Our expression analysis of miR-6240 indicates that it is ubiquitously expressed across various tissues, suggesting that miR-6240 may also play a functional role beyond skeletal muscle. In fact, miR-6240 was reportedly involved in proliferation and mitosis of cardiomyocytes and cardiac regeneration. Significant increases in EdU-positive cells and pH3-positive cells were observed in miR-6240 mimic-treated mouse cardiomyocytes [61]. In addition, transfection with miR-6240-mimic significantly increased human umbilical vein endothelial cells (HUVECs) proliferation, tolerance to H_2_O_2_-induced injury, tube-like structure formation, and cell migration. This implies that miR-6240 may regulate endothelial cell behavior and influence angiogenesis [62]. In this study, we show that *Igf2bp3* expression was downregulated in response to miR-6240 overexpression. It was further verified by the dual luciferase and RIP assays that miR-6240 directly targets *Igf2bp3*. Next, we found that miR-6240 promoted the myoblasts proliferation and inhibited their differentiation and fusion, which showed opposite functions to *Igf2bp3*. Our findings revealed the myogenic regulation role of miR-6240 by targeting *Igf2bp3*.

MYMK is a muscle-specific membrane protein that plays an active role in regulating the fusion of mononuclear myoblasts into multinucleated myofibers [63–65]. We observed a strong positive correlation between the expression levels of *Mymk* and *Igf2bp3*, indicating that *Igf2bp3* might facilitate C2C12 myoblast fusion by modulating *Mymk*. Previous research has demonstrated that MyoD and MyoG are bound to the *Mymk* promoter region, thereby promoting chicken myoblast fusion [7]; miR-140-3p inhibits *Mymk* expression and myoblast fusion by targeting the 3′ UTR region of *Mymk* [7], miR-491 inhibits skeletal muscle differentiation through targeting *Mymk* [12], and miR-205 affects porcine myoblast fusion similarly through *MYMK*, its target gene [57]. In this work, we observed that the knockdown of *Igf2bp3* led to a significant decrease in the expression of *Mymk*, whereas overexpression of *Igf2bp3* resulted in the increase of *Mymk* expression. The *Mymk* expression was significantly downregulated following *Igf2bp3* knockout, but was restored upon rescuing *Igf2bp3*. Moreover, the RIP-qPCR experiment revealed the significantly higher enrichment of *Mymk* in the *Igf2bp3* overexpression group. IGF2BP3 has been documented to enhance target gene mRNA stability in an m^6^A-dependent manner [66]. Furthermore, we performed mRNA stability analysis to confirm this, and found that *Igf2bp3* affects *Mymk* mRNA stability. These findings suggest that *Igf2bp3* increases *Mymk* expression by enhancing *Mymk* mRNA stability, thereby promoting fusion of myoblasts.

## Conclusions

The study elucidated the regulatory roles of *Igf2bp3* in myoblast fusion. We identified miR-6240 as a novel miRNA that involves regulating myogenesis by targeting *Igf2bp3* to suppress its expression. Furthermore, our investigation revealed *Mymk* as a direct target of *Igf2bp3* during the myoblast fusion process. Collectively, our findings underscore the significance of the miR-6240/*Igf2bp3*/*Mymk* axis in myoblast fusion (Graphical Abstract), thereby enhancing our comprehension of the intricate mechanism governing skeletal muscle development.

## Supplementary Information

**Additional file 1**.

## Data Availability

The data used to support the findings of this study are included within the article.

## Abbreviations

CCK-8: Cell counting kit-8
EdU: 5-Ethynyl-2′-deoxyuridine
*Igf2bp3*: Insulin-like growth factor 2 mRNA-binding protein 3
m^6^A: *N*^6^-methyladenosine
*Mymk*: myomaker
*Mymx*: myomixer
RBPs: RNA-binding proteins
RIP: RNA immunoprecipitation
RT-qPCR: Real-time quantitative polymerase chain reaction
