# Supplementary material for: The miR-6240 target gene Igf2bp3 promotes myoblast fusion by enhancing myomaker mRNA stability
Source: Cell Mol Biol Lett. 2024 Dec 5;29:152. doi: 10.1186/s11658-024-00650-1 (PMC11622686; doi:10.1186/s11658-024-00650-1)
Supplement: Supplementary file 1 — Additional file 1. [file 11658_2024_650_MOESM1_ESM.docx]

**The miR-6240 target gene *Igf2bp3* promotes myoblast fusion by enhancing myomaker mRNA stability**

Yuxin Huang^1,2,4^†, Wei Wang^1,3,4^†, Xinhao Fan^1,3,4^, Xiaoqin Liu^1,4^, Weiwei Liu^1,2,4^, Zishuai Wang^1,4^, Yixing Li^2^, Yalan Yang^1,4,^*, and Zhonglin Tang^1,4,^*

**Supplemental information**

**
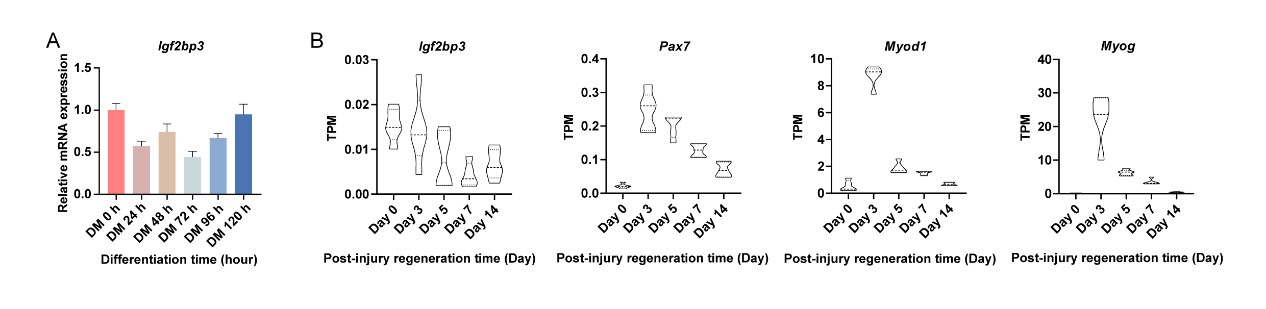
**

**Supplementary Fig. S1. The expression changes of *Igf2bp3* during myoblast differentiation and muscle regeneration. (A)** RT-qPCR analysis of the expression of *Igf2bp3* during C2C12 myoblast differentiation. **(B)** The expression changes of *Igf2bp3*, *Pax7*, *Myod1*, and *Myog* during CTX-induced mouse skeletal muscle regeneration. This data [1] is sourced from https://www.ncbi.nlm.nih.gov/geo/query/acc.cgi?acc=GSE159024. For normalization, *Gapdh* was employed. *N*=3 in each group. Data are represented as mean±SEM.


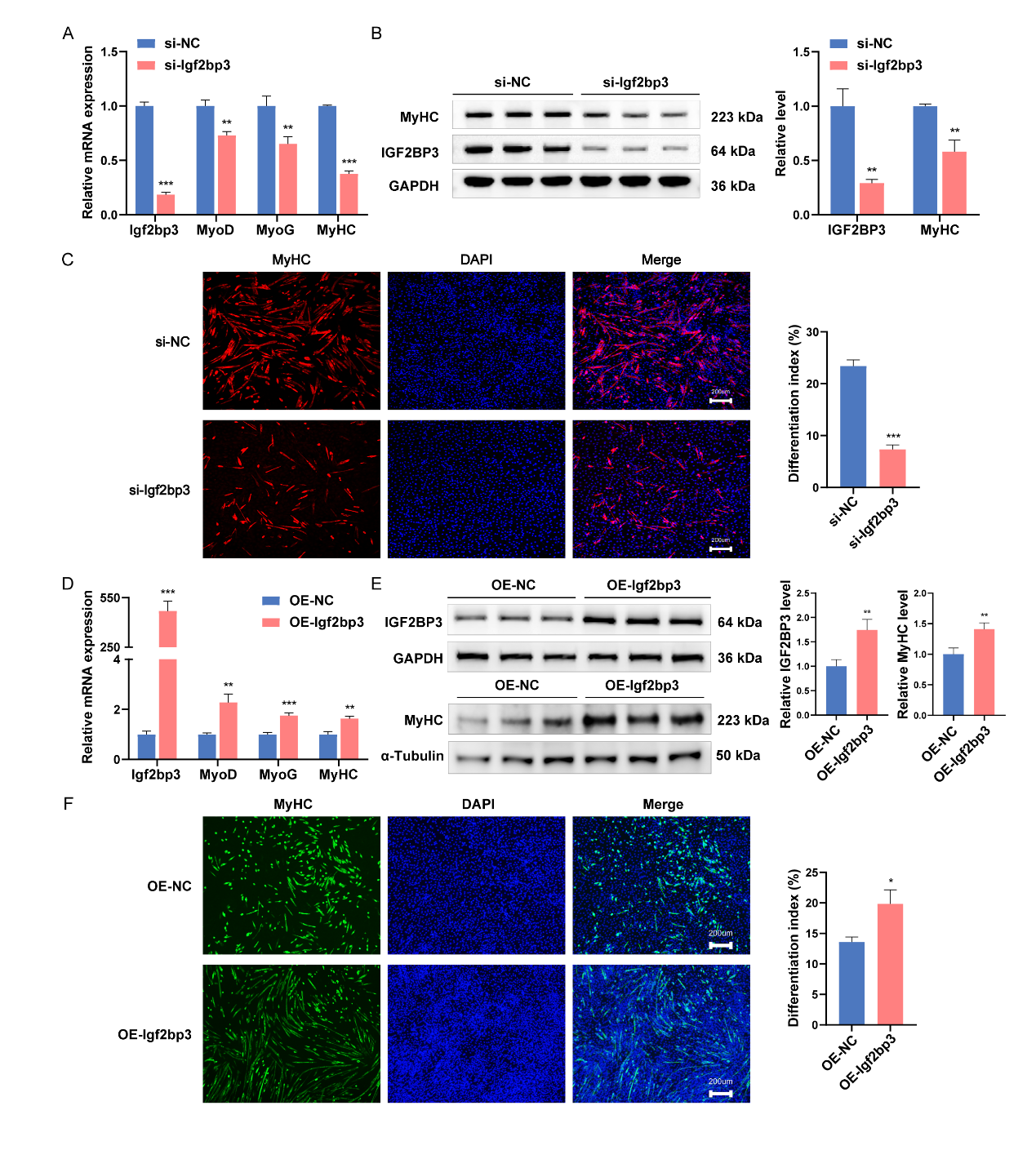


**Supplementary Fig. S2.** **The function of *Igf2bp3* on C2C12 myoblast differentiation. (A, B)** The mRNA (A) and protein (B) levels of *Igf2bp3* and differentiation markers were measured by RT-qPCR and western blot following transfection with *Igf2bp3* siRNA or a negative control; using ImageJ, the protein gray value was calculated. **(C)** MyHC (red) was detected by immunofluorescence on differentiation day 3 in C2C12 myoblasts transfected with *Igf2bp3* siRNA or a negative control. To detect the cell nuclei, DAPI (blue) was utilized. Micrographs were taken using 100× magnification (scale bar, 200 µm). Measurements were made of the differentiation index. **(D, E)** The mRNA (D) and protein (E) levels of *Igf2bp3* and differentiation markers were measured by RT-qPCR and western blot following transfection with pcDNA3.1(+)-*Igf2bp3* or a negative control. The protein gray value was evaluated by ImageJ. **(F)** Immunofluorescence detection of MyHC (green) in C2C12 myoblasts after transfection with pcDNA3.1(+)-*Igf2bp3* or a negative control. The cell nuclei were visible with DAPI (blue). Micrographs were taken using 100× magnification (scale bar, 200 µm). Quantification was performed on the differentiation index. GAPDH or α-tubulin was utilized as an internal control. *N* = 3 in each group. Data are represented as mean ± SEM. **P* < 0.05; ***P* < 0.01; ****P* < 0.001 (Student’s *t*-test).


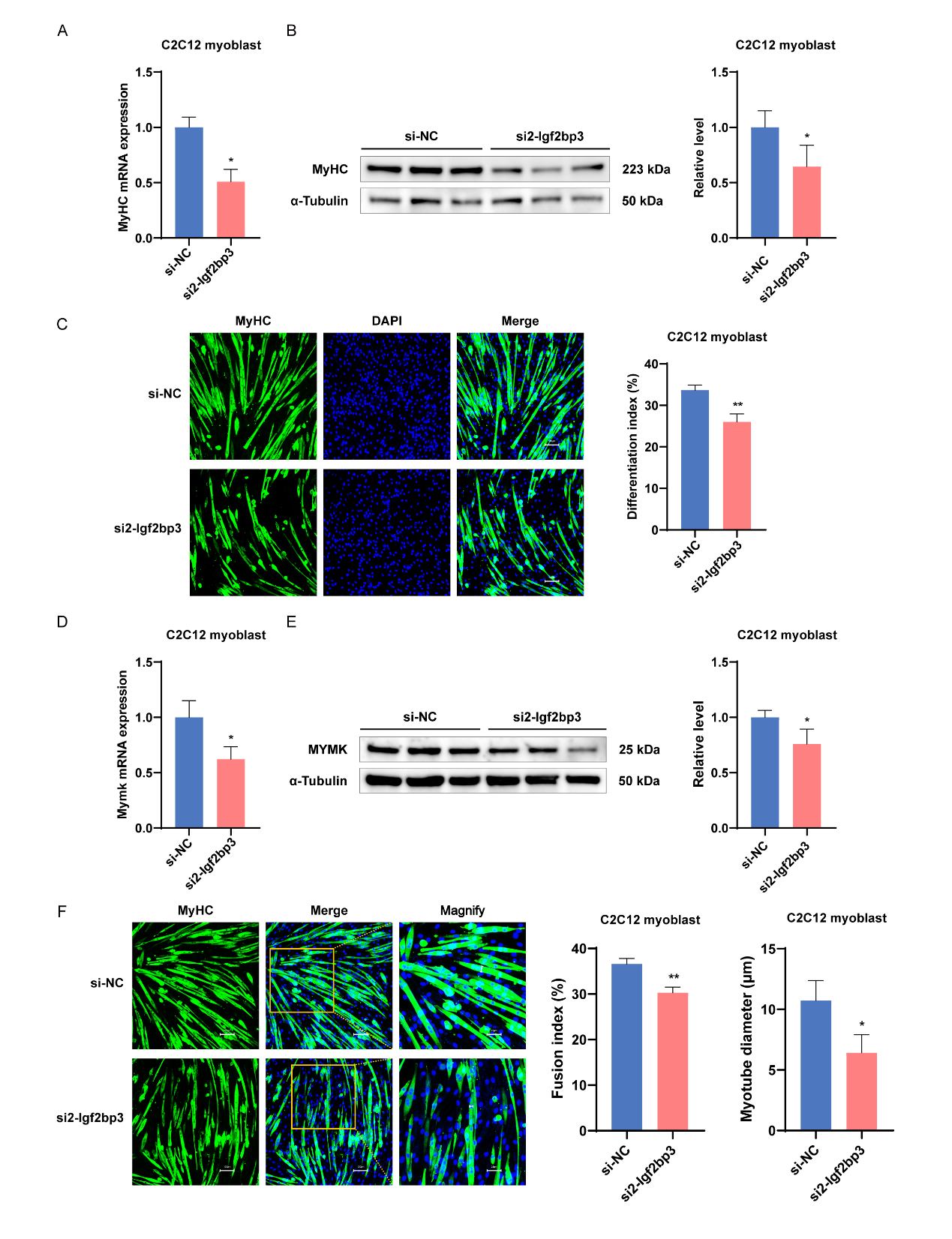


**Supplementary Fig. S3. Effect of transfecting another *Igf2bp3* siRNA on differentiation and fusion of C2C12 myoblasts.** (A, B) mRNA (A) and protein (B) levels of *MyHC* were measured by RT-qPCR and western blot following transfection with *Igf2bp3* siRNA-2 (si2-*Igf2bp3*) or a negative control (si-NC) in C2C12 myoblasts. (C) Immunofluorescence detection of MyHC (green) in C2C12 myoblasts transfected with *Igf2bp3* siRNA-2 or a negative control. To detect cell nuclei, DAPI (blue) was utilized. Measurements were made of the differentiation index. Micrographs were taken using 200× magnification (scale bar, 100 µm). (D, E) The expression levels of *Mymk* were measured by RT-qPCR (D) and western blot (E) after transfecting another *Igf2bp3* siRNA. (F) Immunofluorescence analysis on differentiation day 5 of C2C12 myoblasts after knockdown of *Igf2bp3* by *Igf2bp3* siRNA-2, stained with MyHC (green). The cell nuclei were visible with DAPI (blue). Micrographs of MyHC and merge were taken using 200× magnification (scale bar, 100 µm) and magnified the partial part of the merge image by 4× (scale bar, 25 µm). *Gapdh* or α-tubulin was utilized as an internal control. *N* = 3 in each group. Data are represented as mean ± SEM. **P* < 0.05; ***P* < 0.01 (Student’s *t*-test).


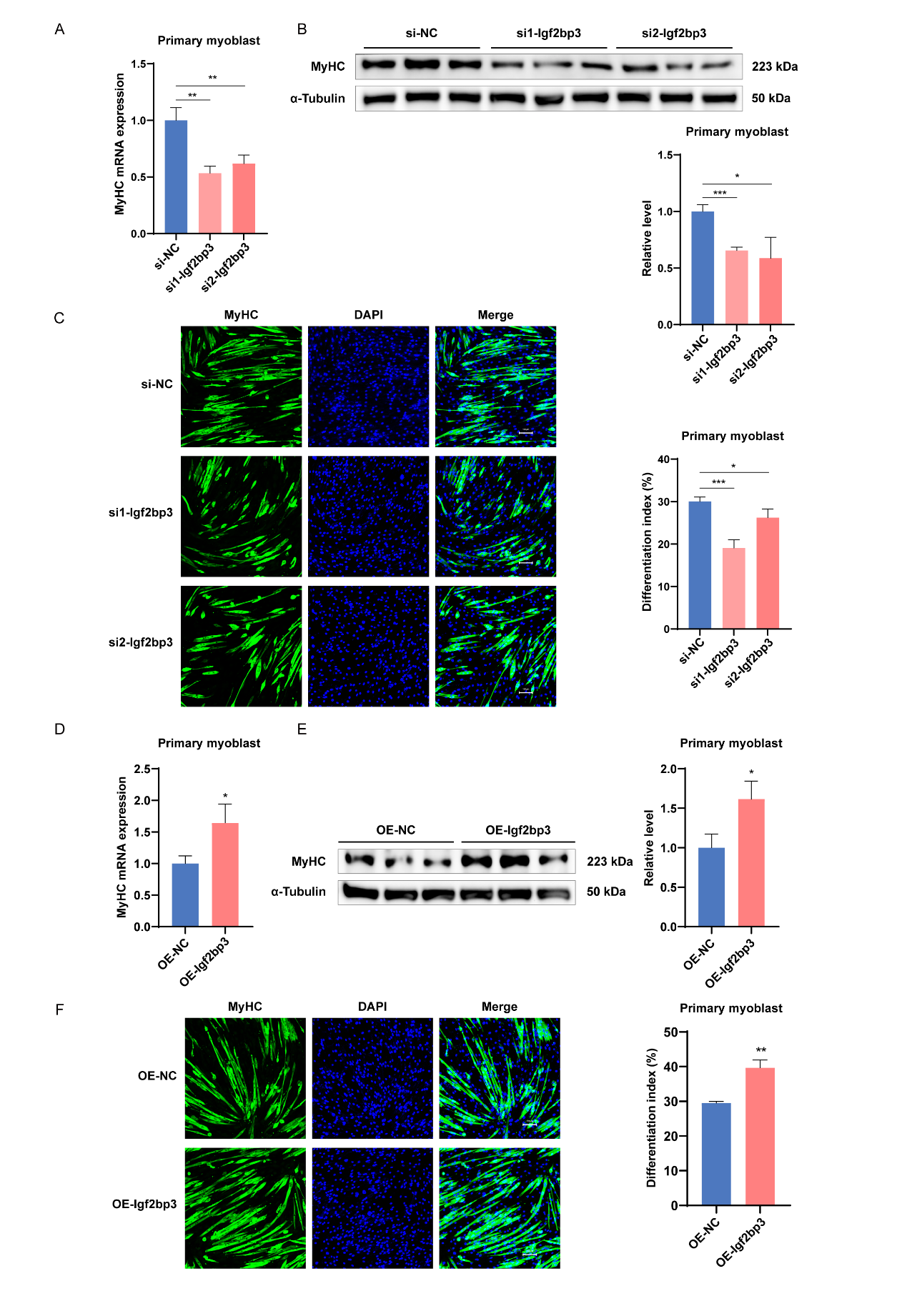


**Supplementary Fig. S4. The function of *Igf2bp3* on primary myoblast differentiation. (A, B)** The mRNA (A) and protein (B) levels of *MyHC* were measured by RT-qPCR and western blot following transfection with *Igf2bp3* siRNA-1 (si1-*Igf2bp3*), *Igf2bp3* siRNA-2 (si2-*Igf2bp3*), or a negative control (si-NC); using ImageJ, the protein gray value was calculated. **(C)** MyHC (green) was detected by immunofluorescence on differentiation day 3 in primary myoblasts transfected with 2 different *Igf2bp3* siRNAs or a negative control. To detect the cell nuclei, DAPI (blue) was utilized. Micrographs were taken using 200× magnification (scale bar, 100 µm). Measurements were made of the differentiation index. **(D, E)** The mRNA (D) and protein (E) levels of MyHC were measured by RT-qPCR and western blot following transfection with pcDNA3.1(+)-*Igf2bp3* or a negative control. The protein gray value was evaluated by ImageJ. **(F)** Immunofluorescence detection of MyHC (green) in primary myoblasts after transfection with pcDNA3.1(+)-*Igf2bp3* or a negative control. The cell nuclei were visible with DAPI (blue). Micrographs were taken using 200× magnification (scale bar, 100 µm). Quantification was performed on the differentiation index. *Gapdh* or α-tubulin was utilized as an internal control. *N* = 3 in each group. Data are represented as mean ± SEM. **P* < 0.05; ***P* < 0.01; ****P* < 0.001 (Student’s *t*-test).


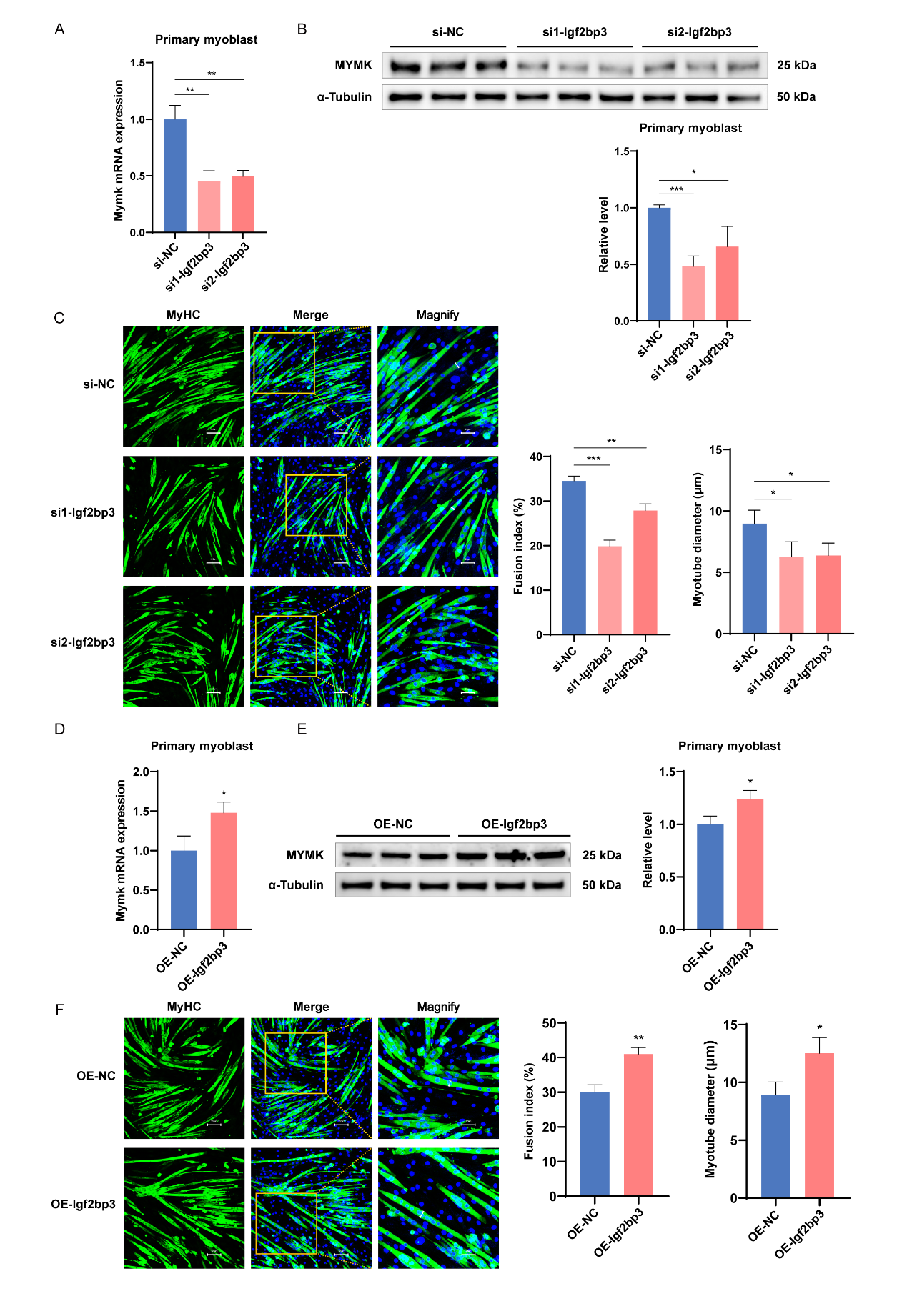


**Supplementary Fig. S5. The function of *Igf2bp3* on primary myoblast fusion. (A, B)** The *Mymk* expression of primary myoblasts was detected by RT-qPCR (A) and western blot (B) following transfection of 2 different *Igf2bp3* siRNAs. **(C)** Primary myoblasts were transfected with 2 siRNAs of *Igf2bp3* or a negative control; their fusion was tracked by measuring the diameter of the myotube, the fusion indices, and the MyHC (green) levels using immunofluorescence. **(D, E)** The expression levels of *Mymk* were measured by RT-qPCR (D) and western blot (E) after overexpression of *Igf2bp3*. **(F)** Immunofluorescence analysis of primary myoblasts after *Igf2bp3* overexpression, stained with MyHC (green). Quantification of the fusion index and myotube diameter was done. For (C) and (F), the cell nuclei were visible with DAPI (blue); micrographs of MyHC and merge were taken using 200× magnification (scale bar, 100 µm) and the partial part of the merge image was magnified by 4× (scale bar, 25 µm). *Gapdh* or α-tubulin was utilized as an internal control. *N* = 3 in each group. Data are represented as mean ± SEM. **P* < 0.05; ***P* < 0.01; ****P* < 0.001 (Student’s *t*-test).


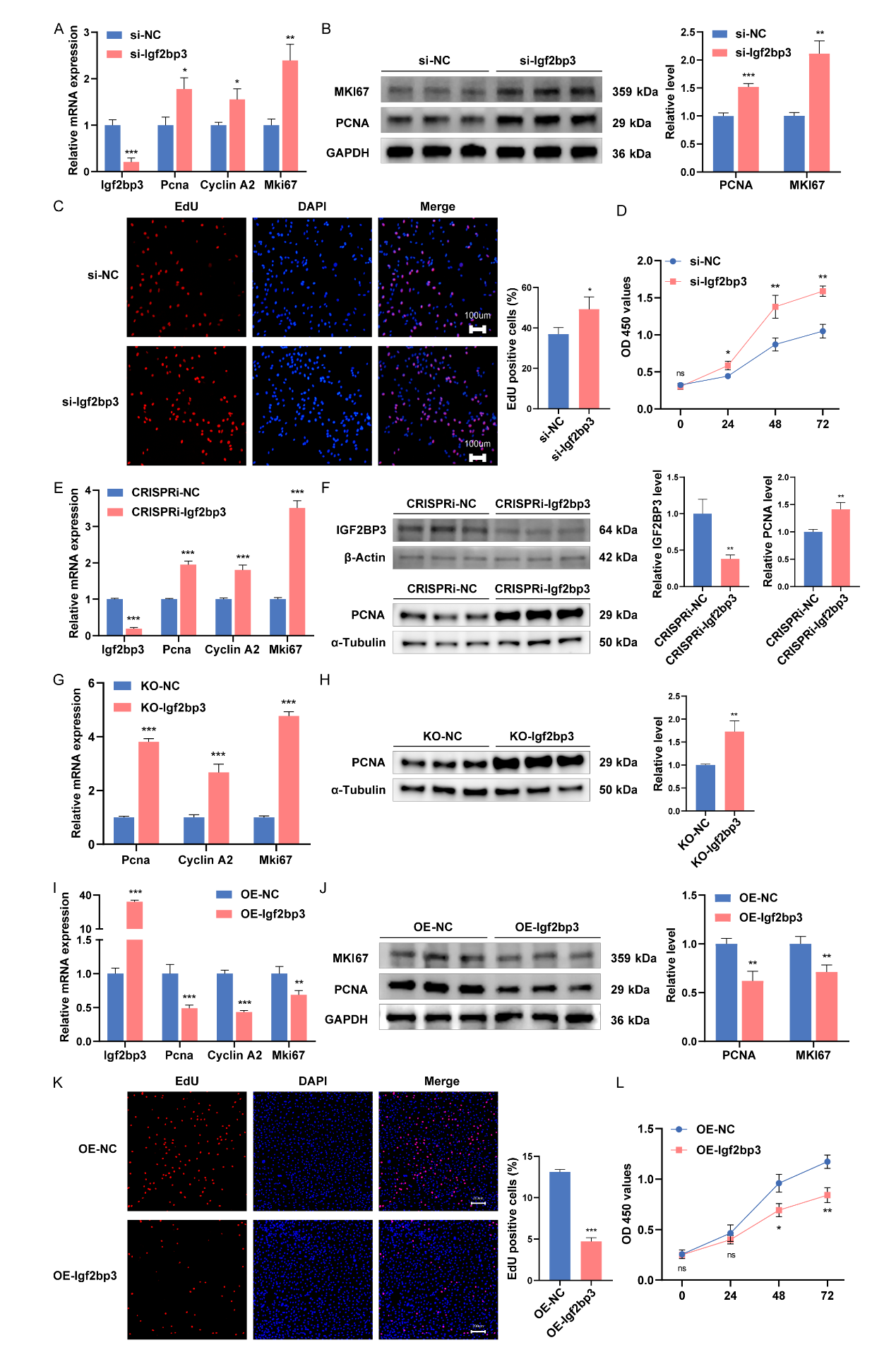


**Supplementary Fig. S6. The function of *Igf2bp3* on C2C12 myoblast proliferation. (A, B)** The mRNA (A) and protein (B) levels of *Igf2bp3* and proliferation markers were measured by RT-qPCR and western blot following transfection with *Igf2bp3* siRNA or a negative control; using ImageJ, the protein gray value was calculated. **(C)** EdU assays of C2C12 myoblasts after transfection with si-*Igf2bp3* or a negative control. Nuclei are shown by DAPI (blue) fluorescence, whereas EdU (red) fluorescence is employed as a proliferation indication. Micrographs were taken using 200× magnification (scale bar, 100 µm). Quantification was made of the EdU-positive cells. **(D)** CCK-8 assay-derived cell growth curves, following transfection with *Igf2bp3* siRNA or a negative control. **(E-H)** Proliferation marker expressions were detected in the CRISPRi-*Igf2bp3* C2C12 cell line (E, F) and the KO-*Igf2bp3* C2C12 cell line (G, H) by RT-qPCR and western blot analysis. **(I, J)** The mRNA (I) and protein (J) levels of *Igf2bp3* and proliferation markers were measured by RT-qPCR and western blot following transfection with pcDNA3.1(+)-*Igf2bp3* or a negative control. **(K)** EdU assays of C2C12 myoblasts after transfection with pcDNA3.1(+)-*Igf2bp3* or a negative control. Nuclei are shown by DAPI (blue) fluorescence, whereas EdU (red) fluorescence is employed as a proliferation indication. Micrographs were taken using 100× magnification (scale bar, 200 µm). Quantification was made of the EdU-positive cells. **(L)** CCK-8 assay-derived cell growth curves, following transfection with pcDNA3.1(+)-*Igf2bp3* or a negative control. GAPDH, β-actin, or α-tubulin was utilized as an internal control. *N* = 3 in each group. Data are represented as mean ± SEM. **P* < 0.05; ***P* < 0.01; ****P* < 0.001; ns, not significant (Student’s *t*-test).


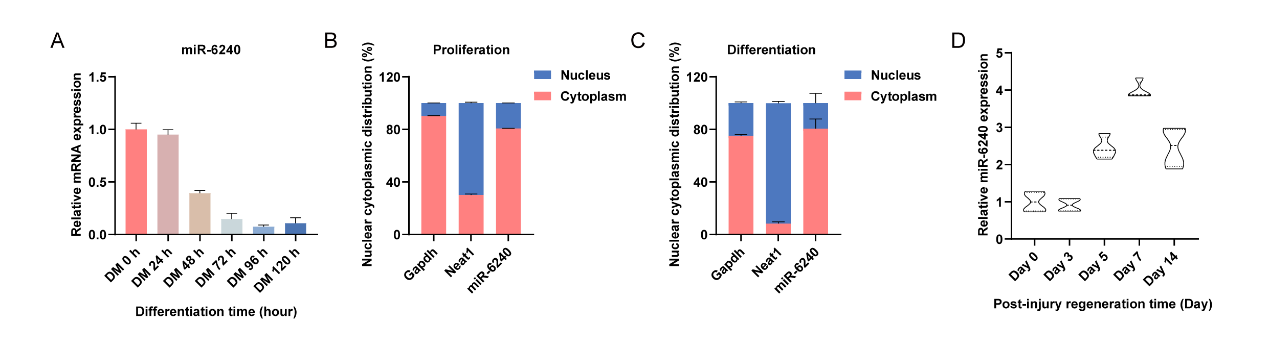


**Supplementary Fig. S7. The expression and cellular localization of miR-6240. (A)** RT-qPCR analysis of the expression of miR-6240 during C2C12 myoblast differentiation. **(B, C)** The distribution of miR-6240 in the nucleus (blue) and cytoplasm (red) was detected by RT-qPCR after purification of nuclear and cytoplasmic RNAs from proliferating (B) and differentiating (C) C2C12 myoblasts. **(D)** RT-qPCR analysis of the expression of miR-6240 during CTX-induced regeneration in TA muscles from C57BL/6 mice. For normalization, *Gapdh* and *Neat1* were employed. *N* = 3 in each group. Data are represented as mean ± SEM.


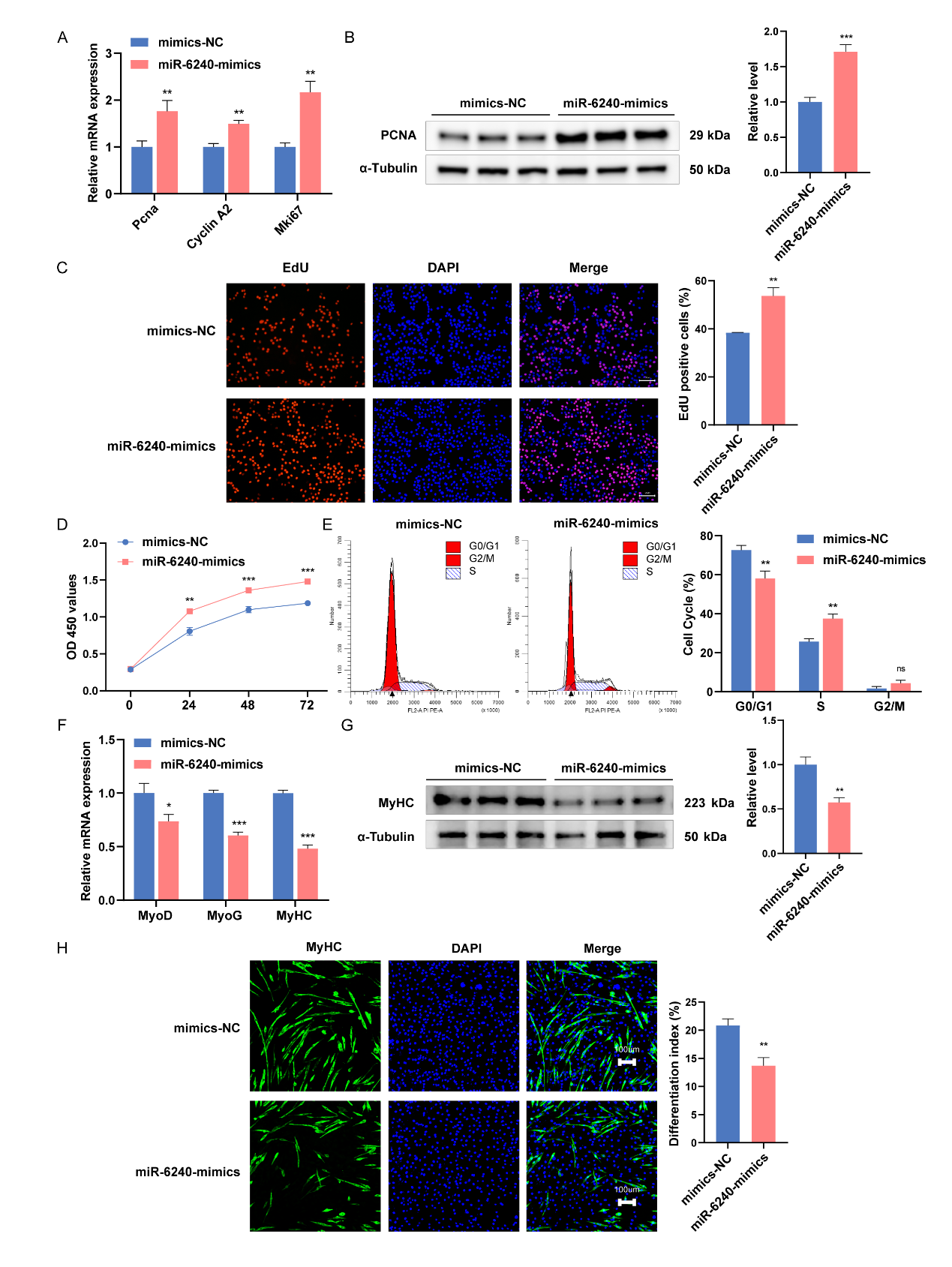


**Supplementary Fig. S8. The function of miR-6240 on C2C12 myoblast proliferation and differentiation. (A, B)** The mRNA (A) and protein (B) levels of proliferation markers were measured by RT-qPCR and western blot following transfection with a control miRNA or miR-6240 mimics, respectively; using ImageJ, the protein gray value was calculated. **(C)** Representative images of EdU assays of C2C12 myoblasts at 36 h after transfection with control miRNA or miR-6240 mimics. EdU (red) fluorescence is used as an indicator of proliferation and nuclei are indicated by DAPI (blue) fluorescence. Micrographs were taken using 200× magnification (scale bar, 100 µm). Quantification was made of the EdU-positive cells. **(D)** CCK-8 assay-derived cell growth curves. **(E)** Analysis of the cell cycle with flow cytometry. **(F, G)** On the third differentiation day following transfection with a control miRNA or miR-6240 mimics, the mRNA (F) and protein (G) levels of differentiation markers were assessed by qPCR and western blot, respectively. **(H)** MyHC-stained (green) C2C12 myoblasts that were induced to differentiate in DM for three days following transfection were examined using immunofluorescence. The cell nuclei were visible with DAPI (blue). Micrographs were taken using 200× magnification (scale bar, 100 µm). Quantification of the differentiation index was done. *Gapdh* or α-tubulin was utilized as an internal control. *N* = 3 in each group. Data are represented as mean ± SEM. **P* < 0.05; ***P* < 0.01; ****P* < 0.001; ns, not significant (Student’s *t*-test).


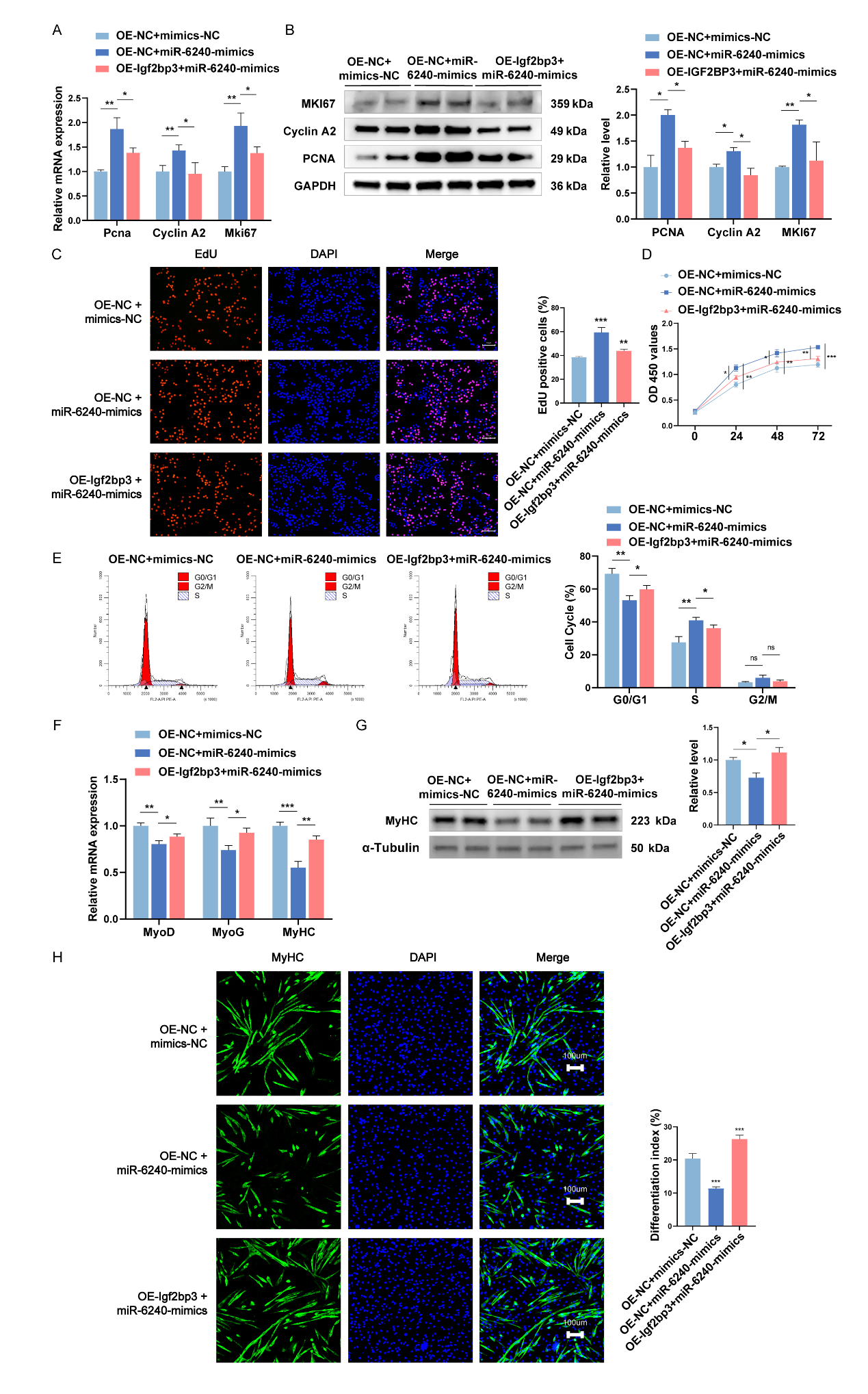


**Supplementary Fig. S9. miR-6240 regulates C2C12 myoblast proliferation and differentiation through targeting *Igf2bp3*.** The C2C12 myoblasts were co-transfected with miRNAs (control miRNA or miR-6240 mimics) and with plasmids (either an empty vector control plasmid or a plasmid expressing *Igf2bp3*). **(A, B)** The mRNA (A) and protein (B) expression of proliferation markers in C2C12 myoblasts, which were collected following transfection. **(C)** An illustration of an EdU assay. EdU (red) fluorescence is used as an indicator of proliferation and nuclei are indicated by DAPI (blue) fluorescence. Micrographs were taken using 200× magnification (scale bar, 100 µm). The EdU-positive cells were counted. **(D)** Cell growth curves were analyzed by CCK-8 assay. **(E)** Investigation of the cell cycle with flow cytometry. **(F, G)** Following transfection, the C2C12 myoblasts were allowed to differentiate for three days before the expression of differentiation markers was assessed by RT-qPCR (F) and western blot (G). **(H)** MyHC-stained (green) C2C12 myoblasts that were induced to differentiate in DM for three days following transfection were examined using immunofluorescence. The cell nuclei were visible with DAPI (blue). Micrographs were taken using 200× magnification (scale bar, 100 µm). The differentiation index was quantified. GAPDH or α-tubulin was utilized as an internal control. *N* = 3 in each group. Data are represented as mean ± SEM. **P* < 0.05; ***P* < 0.01; ****P* < 0.001; ns, not significant (Student’s *t*-test).


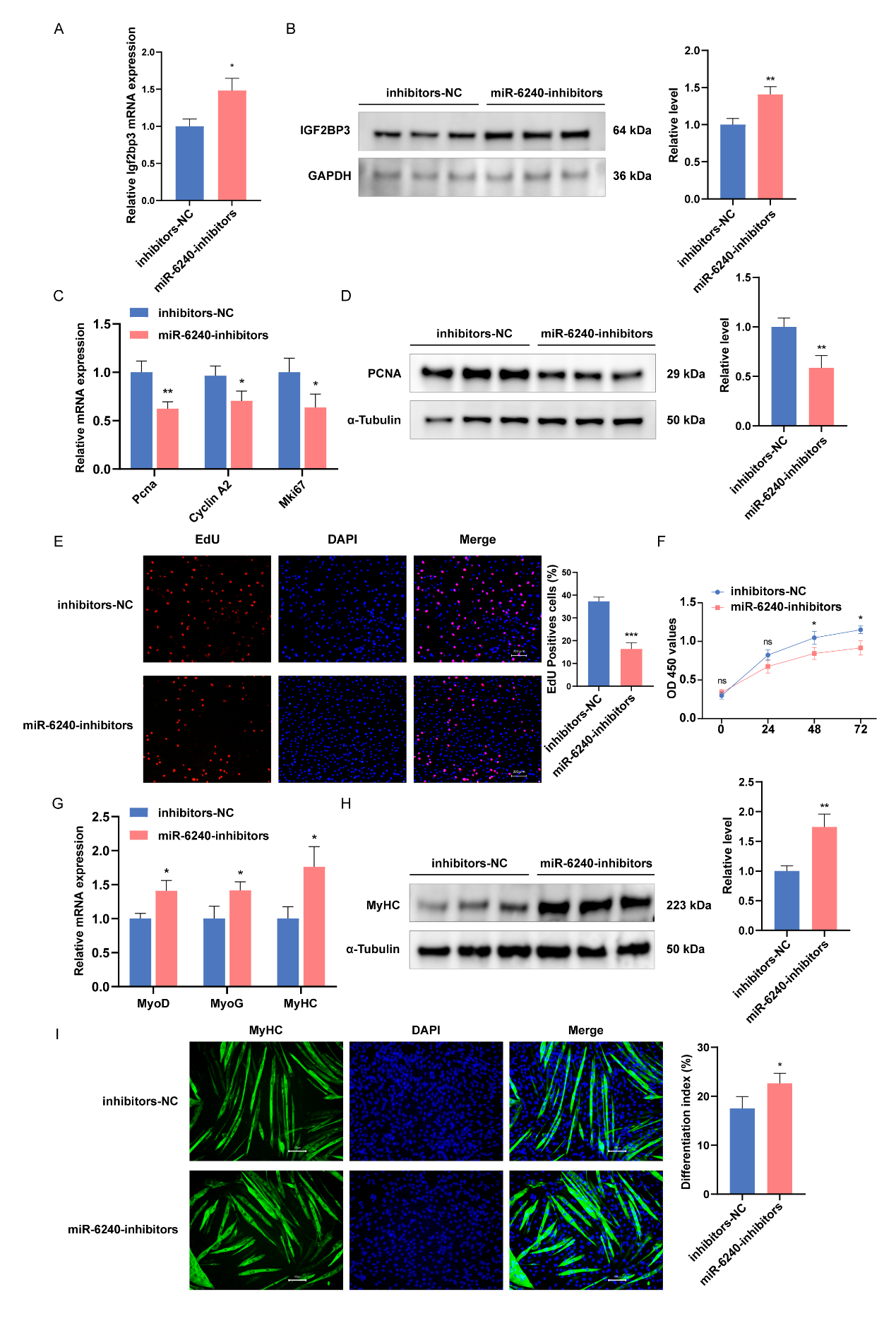


**Supplementary Fig. S10. miR-6240 downregulation inhibits C2C12 myoblast proliferation and promotes differentiation.** **(A, B)** The expression of both *Igf2bp3* mRNA (A) and protein (B) was up-regulated by miR-6240 inhibitors. **(C, D)** The mRNA (C) and protein (D) levels of proliferation markers were measured by RT-qPCR and western blot following transfection with a control miRNA or miR-6240 inhibitors, respectively; using ImageJ, the protein gray value was calculated. **(E)** Representative images of EdU assays of C2C12 myoblasts at 36 h after transfection with control miRNA or miR-6240 inhibitors. EdU (red) fluorescence is used as an indicator of proliferation and nuclei are indicated by DAPI (blue) fluorescence. Micrographs were taken using 100× magnification (scale bar, 200 µm). Quantification was made of the EdU-positive cells. **(F)** CCK-8 assay-derived cell growth curves. **(G, H)** On the third differentiation day following transfection with a control miRNA or miR-6240 inhibitors, the mRNA (G) and protein (H) levels of differentiation markers were assessed by RT-qPCR and western blot, respectively. **(I)** MyHC-stained (green) C2C12 myoblasts that were induced to differentiate in DM for three days following transfection were examined using immunofluorescence. The cell nuclei were visible with DAPI (blue). Micrographs were taken using 200× magnification (scale bar, 100 µm). Quantification of the differentiation index was done. GAPDH or α-tubulin was utilized as an internal control. *N* = 3 in each group. Data are represented as mean ± SEM. **P* < 0.05; ***P* < 0.01; ns, not significant (Student’s *t*-test).


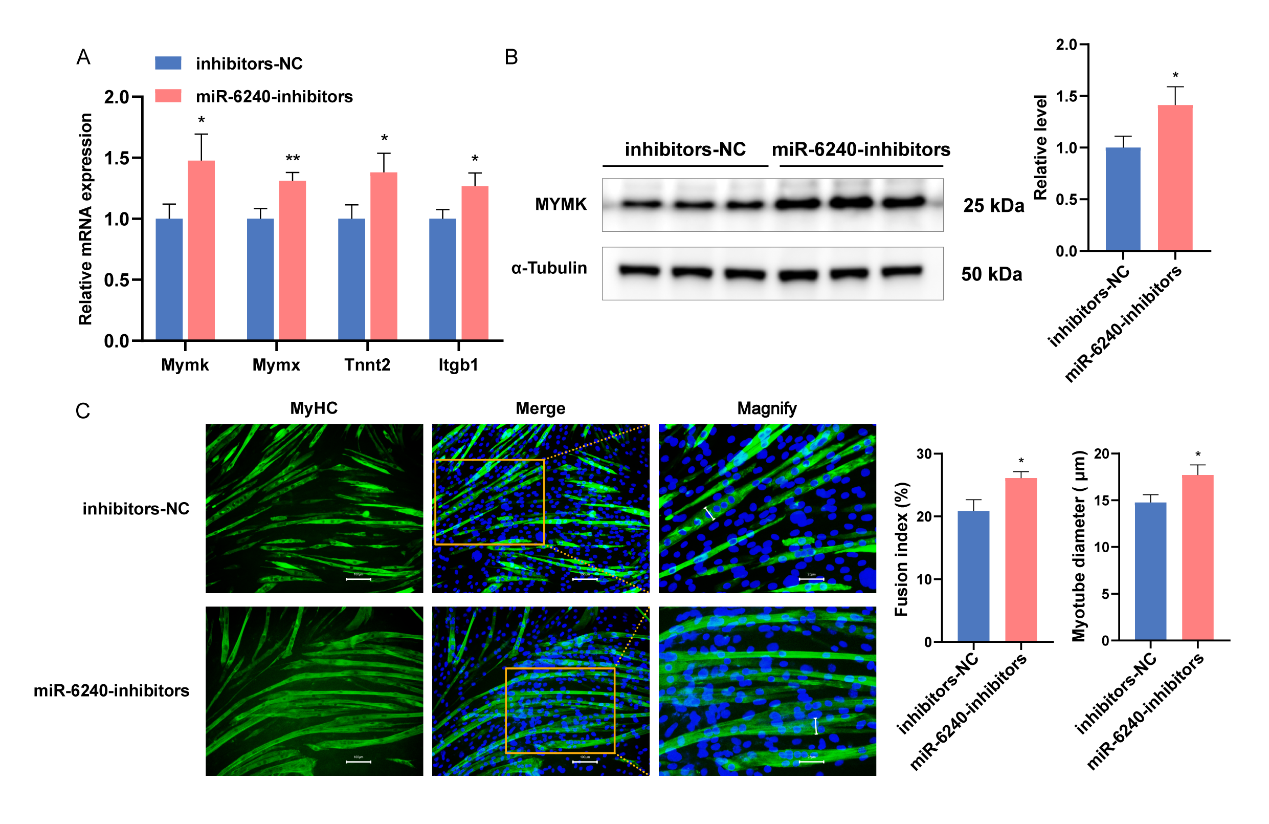


**Supplementary Fig. S11. miR-6240 inhibitors promote C2C12 myoblast fusion.** **(A, B)** After transfection with miR-6240 inhibitors or a negative control, the levels of fusion markers were detected by RT-qPCR (A) and western blot (B). **(C)** C2C12 myoblasts were transfected with miR-6240 inhibitors or a negative control; their fusion was tracked by measuring the diameter of the myotube, the fusion index, and the MyHC (green) levels using immunofluorescence. With the use of DAPI (blue), cell nuclei were observed. Micrographs of MyHC and merge were taken using 200× magnification (scale bar, 100 µm) and a partial part of the merge image was magnified by 4× (scale bar, 25 µm). *Gapdh* or α-tubulin was utilized as an internal control. *N* = 3 in each group. Data are represented as mean ± SEM. **P* < 0.05; ***P* < 0.01 (Student’s *t*-test).

| **Name** | **Sequence (5'-3')** | **Usage** |
| --- | --- | --- |
| OE-Igf2bp3-F | GGGAGACCCAAGCTGGCTAGATGAACAAATTGTACATCGGGAACCT | pcDNA3.1(+) |
| OE-Igf2bp3-R | AACGGGCCCTCTAGACTCGATTACTTCCGCCTTGACTGAGGT | pcDNA3.1(+) |

**Supplementary Table S1. Information regarding the primers used in vector construction.**

| **Name** | **Sequence (5'-3')** | **Usage** |
| --- | --- | --- |
| si-Igf2bp3-1 | CCCACAATTTGAGCAATCA | siRNA |
| si-Igf2bp3-2 | GTCCATTACTATCCTCTCT | siRNA |
| miR-6240-mimics-F | CCAAAGCAUCGCGAAGGCCCACGGCG | mimics |
| miR-6240-mimics-R | CGCCGUGGGCCUUCGCGAUGCUUUGG | mimics |
| miR-6240-inhibitors | CGCCGUGGGCCUUCGCGAUGCUUUGG | inhibitors |

**Supplementary Table S2. Information regarding the siRNA and miRNA mimics and inhibitors used in the present study.**

| **Name** | **Sequence (5'-3')** | **Usage** |
| --- | --- | --- |
| Igf2bp3-F | AACCCTGACTCCTCCCTACC | RT-qPCR |
| Igf2bp3-R | AATCGAAGCTCCCGCAAA | RT-qPCR |
| Neat1-F | GTTCTGGGAGCATCATTCTTT | RT-qPCR |
| Neat1-R | CCTACACCTTACGCAATCTTCT | RT-qPCR |
| MyoD-F | CCACTCCGGGACATAGACTTG | RT-qPCR |
| MyoD-R | AAAAGCGCAGGTCTGGTGAG | RT-qPCR |
| MyoG-F | CCATCCAGTACATTGAGCGCCTACA | RT-qPCR |
| MyoG-R | ACGATGGACGTAAGGGAGTGCAGAT | RT-qPCR |
| MyHC-F | GCCTGGGCTTACCTCTCTATCAC | RT-qPCR |
| MyHC-R | CTTCTCAGACTTCCGCAGGAA | RT-qPCR |
| Mymk-F | CCATCGCTGTGCGGACTT | RT-qPCR |
| Mymk-R | CTCTTTCATCTTCTTCAGCCACTTT | RT-qPCR |
| Mymx-F | CGCCTGCTGCTGCCTGTT | RT-qPCR |
| Mymx-R | GGCTGTTGCTGGCTGAGGAC | RT-qPCR |
| Itgb1-F | TTACAAGAGTGCCGTGACAACTG | RT-qPCR |
| Itgb1-R | GACTAAGATGCTGCTGCTGTGAG | RT-qPCR |
| Tnnt2-F | GCAGAAGAGGTTGGTCCTGATG | RT-qPCR |
| Tnnt2-R | CACCAAGTTGGGCATGAAGAG | RT-qPCR |
| Pcna-F | GGGTGAAGTTTTCTGCAAGTG | RT-qPCR |
| Pcna-R | GTACCTCAGAGCAAACGTTAGG | RT-qPCR |
| Mki67-F | TGCCCGACCCTACAAAATG | RT-qPCR |
| Mki67-R | GAGCCTGTATCACTCATCTGC | RT-qPCR |
| cyclin A2-F | GCCTTCACCATTCATGTGGAT | RT-qPCR |
| cyclin A2-R | TTGCTCCGGGTAAAGAGACAG | RT-qPCR |
| Gapdh-F | AGAACATCATCCCTGCATCC | RT-qPCR |
| Gapdh-R | GGTCCTCAGTGTAGCCCAAG | RT-qPCR |
| β-actin-F | GCCTCACTGTCCACCTTCC | RT-qPCR |
| β-actin-R | AGCCATGCCAATGTTGTCTCTT | RT-qPCR |
| U6-F | CTCGCTTCGGCAGCACA | RT-qPCR |
| U6-R | AACGCTTCACGAATTTGCGT | RT-qPCR |
| miR-6240-RT | GTCGTATCCAGTGCAGGGTCCGAGGTATTCGCACTGGATACGACCGCCGT | RT |
| miR-6240-F | AGCATCGCGAAGGCCC | RT-qPCR |
| miR-320-3p-RT | GTCGTATCCAGTGCAGGGTCCGAGGTATTCGCACTGGATACGACTCGCCC | RT |
| miR-320-3p-F | GCGAAAAGCTGGGTTGAGA | RT-qPCR |
| miR-486a-5p-RT | GTCGTATCCAGTGCAGGGTCCGAGGTATTCGCACTGGATACGACCTCGGG | RT |
| miR-486a-5p-F | CGCGTCCTGTACTGAGCTGC | RT-qPCR |
| miR-486b-5p-RT | GTCGTATCCAGTGCAGGGTCCGAGGTATTCGCACTGGATACGACCTCGGG | RT |
| miR-486b-5p-F | CGCGTCCTGTACTGAGCTGC | RT-qPCR |
| miR-R | AGTGCAGGGTCCGAGGTATT | RT-qPCR |
| Mymk-RIP-F | ACTGGCCGACTTTGATGAACC | RIP-qPCR |
| Mymk-RIP-R | CCACTTTACAGCAATGATGA | RIP-qPCR |

**Supplementary Table S3. Information regarding the primers used in the present study.**

**Reference**

1. Maehara K, Tomimatsu K, Harada A, Tanaka K, Sato S, Fukuoka M, et al. Modeling population size independent tissue epigenomes by ChIL-seq with single thin sections. Molecular systems biology. 2021;17(11):e10323.
